# Approaches toward the Separation, Modification, Identification and Scale up Purification of Tetracyclic Diterpene Glycosides from *Stevia rebaudiana* (Bertoni) Bertoni

**DOI:** 10.3390/molecules26071915

**Published:** 2021-03-29

**Authors:** Wilmer H. Perera, James D. McChesney

**Affiliations:** 1 CAMAG Scientific, Inc., 515 Cornelius Harnett Drive, Wilmington, NC 28401, USA; 2 Ironstone Separations, Inc., Etta, MS 38627, USA

**Keywords:** *Stevia rebaudiana*, HPTLC, HPLC methods, mass spectrometry dissociation pattern, NMR, endocyclic and exocyclic diterpene glycosides, scale up

## Abstract

*Stevia rebaudiana* (Bertoni) Bertoni is a plant species native to Brazil and Paraguay well-known by the sweet taste of their leaves. Since the recognition of rebaudioside A and other steviol glycosides as generally recognized as safe by the United States Food and Drug Administration in 2008 and grant of marketing approval by the European Union in 2011, the species has been widely cultivated and studied in several countries. Several efforts have been dedicated to the isolation and structure elucidation of minor components searching for novel non-caloric sugar substitutes with improved organoleptic properties. The present review provides an overview of the main chemical approaches found in the literature for identification and structural differentiation of diterpene glycosides from *Stevia rebaudiana*: High-performance Thin-Layer Chromatography, High-Performance Liquid Chromatography, Electrospray Ionization Mass Spectrometry and Nuclear Magnetic Resonance Spectroscopy. Modification of diterpene glycosides by chemical and enzymatic reactions together with some strategies to scale up of the purification process saving costs are also discussed. A list of natural diterpene glycosides, some examples of chemically modified and of enzymatically modified diterpene glycosides reported from 1931 to February 2021 were compiled using the scientific databases Google Scholar, ScienceDirect and PubMed.

## 1. Introduction

*Stevia rebaudiana*, a native plant species to Brazil and Paraguay, was discovered by Mosè Giacomo Bertoni in 1899, an Italian-speaking Swiss naturalist and described as *Eupatorium rebaudianum* Bertoni. In 1905 the plant was grouped in a different genus and named as *Stevia rebaudiana* (Bertoni) Bertoni. *S. rebaudiana* has been widely used to sweeten teas and potions for centuries by Guarani indigenous due to the high content in diterpene glycosides (DGs) in their leaves [1]. Essential oils have been reported from *S. rebaudiana*, being spathulenol (13.4–40.9%), caryophyllene oxide (1.3–18.7%), β-caryophyllene (2.1–16.0%) and β-pinene (5.5–21.5%) the major volatile compounds [2]. Non-glycosidic labdane-type diterpenes have also been isolated e.g., sterebins I -N [3] and several hydroxycinnamate and flavonoid derivatives have been identified [4,5]. There is no doubt, that the DGs is the most significant group of secondary metabolites from *S. rebaudiana* due to their applications in the Sweeteners Industry. Stevioside was the first steviol glycoside isolated from this Asteraceae in 1931, reported by M. Bridel and R. Lavielle, two French Chemist [6]. Then, rebaudiosides A, B and steviolbioside were discovered by a Japanese group 45 years later, followed by dulcosides A, B and rebaudiosides C (same chemical structure as dulcoside B), D and E in 1977 [7,8,9,10]. In 2002 rebaudioside F and its saponification products were reported [11]. In 2008, the United States Food and Drug Administration granted generally recognized as safe (GRAS) regulatory acceptance of rebaudioside A while Food Standards Australia New Zeeland accepted the use of steviol glycosides [12,13]. Other steviol glycosides were recognized as safe in USA in 2010, and the European Union approved DGs from *S. rebaudiana* for marketing in November 2011 [14,15]. A great scientific contribution was made by Ohta et al. [16], where 21 steviol glycosides were isolated and characterized. Since that time, multiple groups have been working intensively on searching for new steviol glycosides. Rebaudioside A and stevioside, the major steviol glycosides (accounting for more than 40% of the DGs present in leaf extract) have shown more potent natural sweetness than sucrose which make them good candidates as non-caloric sugar-substitutes [1]. Hence, *S. rebaudiana* is being widely commercialized in mainland China, Japan, Korea, India, and elsewhere (Midmore and Rank, 2012) and several rebaudioside A-based products as well as table-top sweeteners are commercially available in the US consumer market.

It was recently discovered that rebaudioside A and stevioside, potentiate the activity of TRPM5 (a Ca^2+^-activated cation channel expressed in type II taste receptor cells and pancreatic β-cells) enhancing glucose-induced insulin secretion in a TRPM5-dependent manner. TRPM5 has been suggested as a potential target to prevent and treat type 2 diabetes [17]. It has also been described that steviol glycosides stimulates insulin secretion in a dose- and glucose-dependent manner from isolated mouse islets of Langerhans. Steviol glucuronide has been identified in human urine, thus steviol is the main active metabolite after oral intake of steviol glycosides [18,19]. The antihyperglycemic and blood pressure-reducing effects of stevioside in the diabetic Goto-Kakizaki rat and the mechanism of the hypoglycemic effect of stevioside has been described [20,21].

Despite the potential positive human health benefits of rebaudioside A and the relatively easy access to this steviol glycoside, its lingering aftertaste makes it less desirable as a sweetener. Therefore, several efforts have been dedicated to preparing commercial *Stevia* extracts rich in DGs to find next generation non-caloric DGs with improved organoleptic properties and thus, attract consumers to a better health lifestyle.

Important advances have been made over the last several years in the methods of analysis and purification of these complex mixtures of glycosides. All these developments have allowed the discovery of several new natural and slightly modified DGs with remarkable potential as sugar substitutes e.g., several rebaudioside A isomers, a stevioside like-compound with a disaccharide linked at position C-12 of the aglycone [22], rebaudioside R (unique example with a non-glucose monosaccharide linked directly at position C-13 (xylose) [23], rebaudioside U and similar compounds, the only steviol glycosides with an arabinopyranosyl moiety [24,25], rebaudioside W (unique hexa-glycoside from *S. rebaudiana* with three different sugar monosaccharides attached (glucose, xylose and rhamnose) [26]. Additionally, rebaudioside IX series are the largest natural DGs composed of nine glucose units linked to steviol [27,28,29], only one natural example of rebaudioside M isomer has been reported [30] and a few new compounds with an *ent*-atisene core have also been reported [31,32]. Nowadays, many natural, chemically, and enzymatically modified diterpene glycosides with diverse sugar interlinkages and cores have been discovered, hence, a wide spectrum of structures are now available to evaluate for their sweetener potential and to understand better the relationship between structure-sweetness/lingering aftertaste. Rebaudioside M, has been pointed out as the next generation non-caloric sweetener but in our non-professional tasters experience there are other DGs with interesting organoleptic properties that should be explored e.g., rebaudiosides U and Y.

Herein, we summarize the most noteworthy chemical approaches for the separation, identification and structural elucidation of diterpene glycosides from *Stevia rebaudiana*: High-performance Thin-Layer Chromatography, High-Performance Liquid Chromatography, High-Resolution Electrospray Ionization Mass Spectrometry and Nuclear Magnetic Resonance Spectroscopy. The modification of diterpene glycosides by chemical and enzymatic reactions together with some strategies to achieve the scale up of the purification process economically sustainable are also discussed. An updated listing of the natural and chemically modified diterpene glycosides has been included.

## 2. Results

### 2.1. Chemical and Enzymatic Reactions of Diterpene Glycosides

#### 2.1.1. Alkaline Hydrolysis

*Stevia* DGs are thermostable natural compounds in a neutral solution, although in an alkaline medium with some increase in temperature can be hydrolyzed and yield the free acid compounds at C-19 [16,26].

DGs with one sugar and few examples with two sugars (less hindered as rebaudiosides I and U) attached at position C-19 cleave using mild alkaline conditions. However, DGs with a hindered disaccharide or longer oligomers at C-19 only cleave using stronger alkaline conditions. This difference in saponification conditions for DGs together with the preparation and characterization of some saponified DGs has been useful for the development of a simple and rapid reversed-phase C-18 high-performance liquid chromatography method (HPLC) to identify known and detect novel C-13 oligomer arrangements, see Section 4.1 [26]. In Table 1 are shown all the DGs from *S. rebaudiana* that have been modified by using alkaline conditions.

#### 2.1.2. Acid Hydrolysis

**Figure 1:**
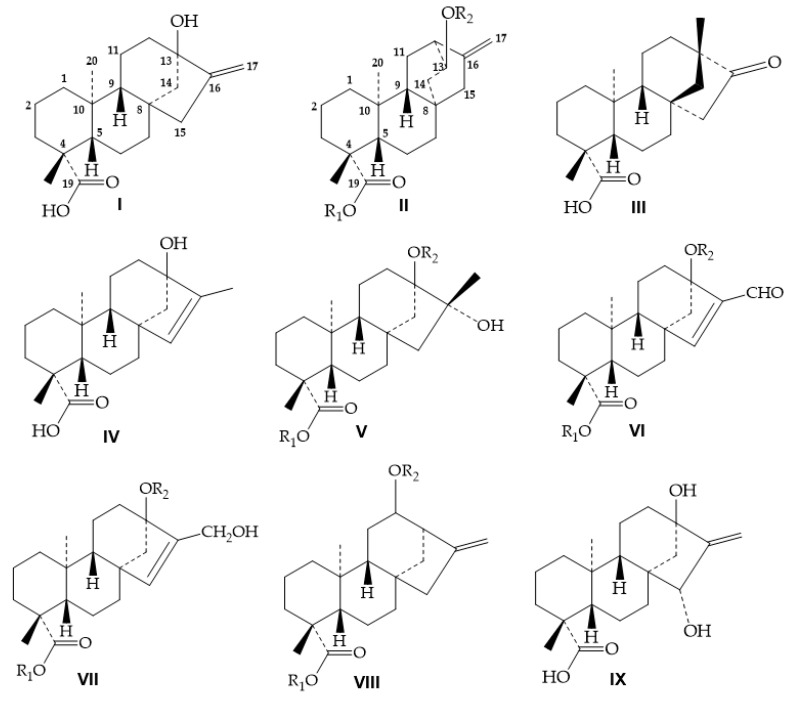
Aglycone cores reported from *S. rebaudiana* as natural forms, degradation products or forming part of a glycoside compound (R_1_ and R_2_). steviol (**I**); *ent*-atisene (**II**); isosteviol (**III**); endo steviol (**IV**); CH_3_ and OH at C-16 (**V**); CHO at C-16 (**VI**); CH_2_OH at C-16 (**VII**); C-12 linkage (**VIII**); 15-α-hydroxy-rebaudioside M (**IX**).

The DGs under acidic conditions may undergo double bond isomerization, sugar cleavage from the glycosides, and Wagner-Meerwein rearrangement of the diterpene aglycone portion depending on temperature. The acid hydrolysis of DGs has been studied since several soft drinks and juices prepared with DGs from *S. rebaudiana* as sweeteners can produce undesirable products under inappropriate temperature storage that may affect the taste of these acidic beverages. Even under different mild acid conditions a double bond isomerization process occurs yielding some by-products that have been isolated and characterized [33,34,35,36,37,38,39]. However, proper high-resolution analytical methods need to be developed to differentiate natural DGs from their endocyclic isomer compounds since they elute very closely in HPLC [40]. Additionally, a cleavage of all the sugars in DGs occurs under strong acid conditions producing a mixture of at least three main aglycones from this reaction: isosteviol (major), the endo steviol isomer and steviol (as very minor component). Hence, acid hydrolysis is not a good method to produce steviol in quantities. Recently, these three structurally close aglycones have been purified in their intact form by a two-step gradient high-performance silica gel chromatography [38]. In Table 1 are shown all the DGs formed under basic or acidic conditions while all the aglycones produced from steviol glycosides under acidic conditions and others reported from *S. rebaudiana* are compiled in Figure 1.

#### 2.1.3. Hydrogenation of the Exocyclic Double Bond

**Figure 2:**
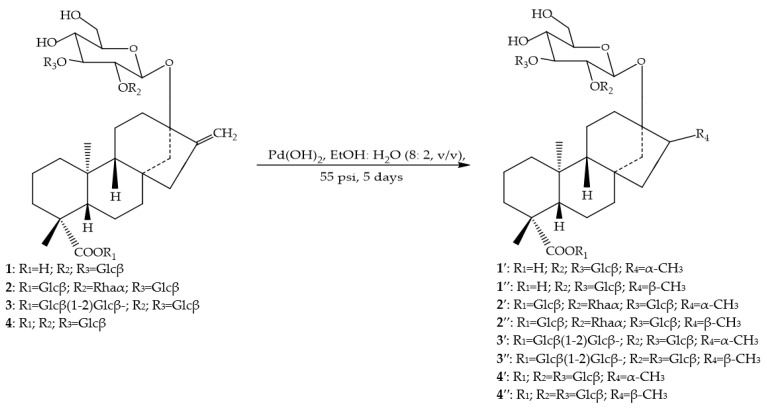
Catalytic hydrogenation of the exocyclic double bond in some steviol glycosides.

Catalytic hydrogenation of the exocyclic olefinic bond has also been studied in detail for some DGs and the sensory evaluation of their derivatives has also been reported. In this reaction, both isomers, α-CH_3_ and β-CH_3_ at position C-16 of the aglycone have been obtained and characterized Figure 2.

Some diterpene glycoside compounds have been prepared from rebaudiosides A, B, C and D using different catalysts and reaction conditions [43,44]. The organoleptic properties of the mixture of the reduced α-CH_3_ and β-CH_3_ diterpene glycoside isomers have been assayed. The reduction of the exocyclic olefinic bond at C-16 decreased the sweet taste of the natural DGs from 25 to 75%, hence the reductive modification of this site of the aglycone steviol is not a good target for improving the organoleptic properties of natural DGs.

#### 2.1.4. Enzymatic Reactions

Steviol glycosides and gibberellins share the first steps of the biosynthetic pathway where the pathway splits after the formation of *ent*-kaurenoic acid to yield gibberellins and steviol. Steviol glycosides are formed after glycosylation by means of the Uridine 5′-diphospho-glycosylltransferase enzymes producing glucosylation, xylosylation, rhamnosylation, etc. in different positions contributing to the formation of wide spectrum in monosaccharide arrangements [45,46].

The preparation of known and new DGs by using glycosyltransferase enzymes and/or genetically modified microorganisms (e.g., *Saccharomyces cerevisiae* strains) have been used as an approach for producing DGs with improved taste. Glycosyltransferase enzymes transfer glycosyl moieties from an activated nucleoside monosaccharide into mono or oligosaccharide acceptor containing molecules. Some glycosyltransferase enzymes retain or invert the stereochemistry of the substrates. Some scientific articles have been recently published and various patents have been granted.

Some of the examples found in the literature retaining the stereochemistry of the substrate are the bioconversion of rebaudioside A into rebaudioside I and M2 (rebaudioside M isomer with Glcβ(1-6)[Glcβ(1-2)]Glcβ_1_- at C-19) and the bioconversion of rebaudioside D3 into (Rebaudioside D isomer with Glcβ(1-6)[Glcβ(1-2)]-Glcβ_1_- at C-13) from rebaudioside E employing UDP-glycosyltransferases using uridine 5-diphosphoglucose (UDP-glucose) as a donor of the sugar moiety.

In the same way, some new minor DGs with inversion of the stereochemistry of some anomeric protons were isolated from a cyclodextrin glycosyltransferase glucosylated *Stevia* extract containing a high percentage of DGs. Interestingly, α-glucosyl linkages were mainly detected at position 4 of some sugar monosaccharide of moieties attached at position C-13 and/or C-19 of the steviol aglycone: 13-[(2-*O*-β-D-glucopyranosyl-3-*O*-(4-*O*-α-D-glucopyranosyl)-β-D-glucopyranosyl-β-D-glucopyranosyl)oxy] ent-kaur-16-en-19-oic acid-[(4-*O*-α-D-glucopyranosyl-β-D-glucopyranosyl) ester]; 13-[(2-*O*-β-D-glucopyranosyl-β-D-glucopyranosyl)oxy] ent-kaur-16-en-19-oic acid-[(4-*O*-(4-*O*-(4-*O*-α-D-glucopyranosyl)-α-D-glucopyranosyl)-α-D-glucopyranosyl)-β-D-glucopyranosyl ester]; 13-[(2-*O*-β-D-glucopyranosyl-3-*O*-(4-*O*-(4-*O*-(4-*O*-α-D-glucopyranosyl)-α-D-glucopyranosyl)-α-D-glucopyranosyl)-β-D-glucopyranosyl-β-D-glucopyranosyl)oxy] ent-kaur16-en-19-oic acid β-D-glucopyranosyl ester and 13-[(2-*O*-β-D-glucopyranosyl-3-*O*-(4-*O*- (4-*O*-(4-*O*-α-D-glucopyranosyl)-α-D-glucopyranosyl)-α-D-glucopyranosyl)-β-D-glucopyranosyl-β-D-glucopyranosyl)oxy] ent-kaur-16-en-19-oic acid-[(4-*O*-α-D-glucopyranosyl-β-D-glucopyranosyl) ester]. The different enzymes, cyclodextrin glycosyl transferase, α and β-Glucosidases, α and β-Galactosidase and β-fructosidase transglycosylation systems together with β-Glycosyltransferase glycosylation systems using UDP-sugars used in the biotransformation of steviol glycosides have been summarized [47].

Additionally, enzymatic hydrolysis seems to be the most efficient method to produce steviol in quantities. Several enzymes have been used for this purpose e.g., juices of the snail *Helix pomatia*, pectinase or hesperidinase affords the aglycone, steviol [6,48,49].

## 3. Methods of Separation for Diterpene Glycosides

### 3.1. Analytical Methods

#### 3.1.1. High-Performance Thin-Layer Chromatography

**Figure 3:**
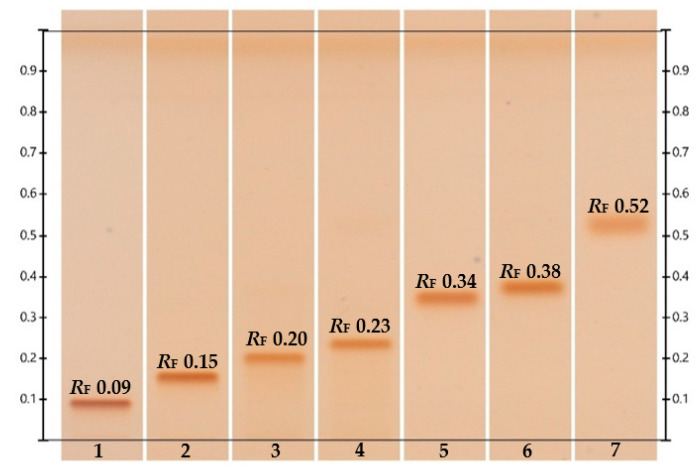
Silica gel HPTLC plate for rebaudiosides D, A, C, stevioside, rebaudioside B, dulcoside A and steviolbioside (tracks 1 to 7). Developed with ethyl acetate, methanol and formic acid (93:40:1 *v*/*v*/*v*) with no saturation but activated with MgCl_2_ at a relative humidity of 33%. The detection was performed after derivatization with a solution of 2-naphthol under white light.

High-Performance Thin-Layer Chromatography (HPTLC) is a standardized methodology that has been widely used in the Botanical Industry [50] but has also found multiple applications in different fields [51,52,53]. Several HPTLC methods have been developed to identify or quantify selected steviol glycosides (stevioside, steviolbioside, dulcoside A and rebaudiosides A–D) in food and Stevia formulations, and also to detect degradation products like steviol and isosteviol [54]. The main stationary phases used have been silica gel 60, silica gel 60 diol, LiChrospher silica gel 60 F254 S and Proteochrom although the best separation has been achieved on HPTLC silica gel 60 plates. Multiple mobile phases have been assessed to optimize the separation of selected steviol glycosides but ethyl acetate, methanol and formic acid (93:40:1 *v*/*v*/*v*) and ethyl acetate, methanol and acetic acid (3:1:1, *v*/*v*/*v*) seem to be the mixtures where the best separation has been achieved so far. However, with these mobile phases the migration of very polar steviol glycosides like rebaudioside M, N and O is not guaranteed and the separation of the endocyclic steviol isomer, a degradation product formed under acidic conditions from steviol glycosides, needs to be further studied. Steviol glycosides have been visualized on the silica plates by derivatization mainly with a solution of 2-naphthol or primuline for aglycones. Hence, densitometric analysis using absorption and fluorescence modes have been used [54,55] for quantitation. Steviol glycosides can be identified by HPTLC-ESI-MS using a TLC-MS interface and specific structural information can be obtained if collision energy is carefully studied as discussed in 4.3. In Figure 3 the HPTLC separation of selected steviol glycosides is presented.

#### 3.1.2. High-Performance Liquid Chromatography

High performance liquid chromatography (HPLC) is the usual methodology for the analyses of DGs with UV, PDA and MS as the most common detectors [56,57]. Several methods have been developed to separate DGs from complex *S. rebaudiana* extract mixtures. Since the most common aglycone is steviol, novel potential sugar-substitutes could be found based on the number of monosaccharides, type of sugar units and their arrangements. These slight structural differences often cause co-elution of DGs in a single HPLC method.

**Figure 4:**
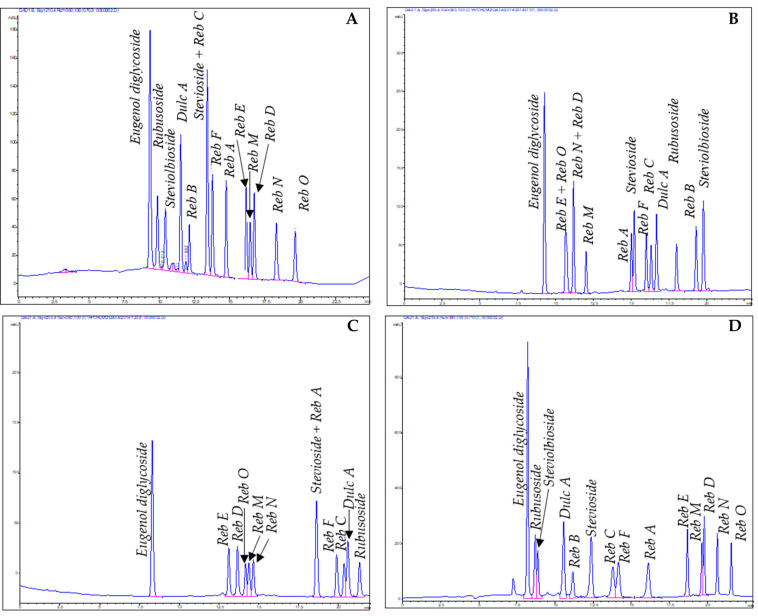

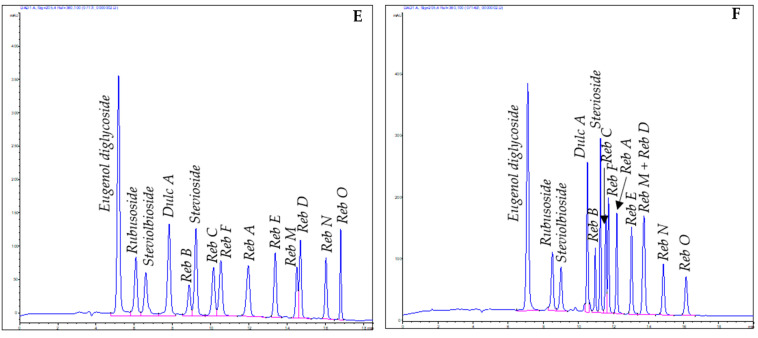
Selected HPLC methods used for the analysis of steviol glycosides: silica method (**A**), RP-C18 (**B**), Synergi (**C**), amino (**D**), HILIC (**E**) and Sepax-diol (**F**).

An overview of the advantages and disadvantages of some HPLC methods using different stationary phases (HILIC, NH_2_, RP-C18, Sepaxdiol, Synergi, silica gel) is given herein, although detailed information can be found [40,58]. Several purified DGs containing different numbers of sugars and linkage arrangements to steviol were analyzed for this purpose. Interestingly, in all the methods reported, acetonitrile: water acidified elution gradients were described for the analyses of DGs [40,58]. The use of a common binary mobile phase is a practical methodology for the analyses of DGs with simple changes in the column adsorbent chemistry (Figure 4). There is no change in selectivity between the silica gel and the amino method. In silica gel, stevioside and rebaudioside C coelute while in an amino column, a good separation is achieved, but rubusoside and steviolbioside are better resolved in silica than in amino column. When a Synergi column is utilized, there is a big room for the separation of very polar steviol glycosides (more polar than rebaudioside O) and also between rebaudiosides A and N. There is no change in selectivity if HILIC column is compared with Sepax-diol one, although sharper peaks are gotten with a Sepax-diol column. The appropriate combination of HPLC methods which best resolved components in the range of retention times of interest give a good understanding of the DGs in the extract and chromatography fractions and provide guidance for further preparative methods to be used.

### 3.2. Preparative Methods

Several approaches have been described in the literature to isolate and purify known and novel DGs. However, most of the preparative methods described allowed the purification of some few milligrams of DGs. Since novel DGs with refined organoleptic properties need to be found to enhance *Stevia* DGs consumption as a non-caloric sweetener and thus improve human health, the purification process of DGs needs to be scaled-up to access for assay the very minor glycosides as potential sugar-substitutes still undiscovered. Herein we describe some strategies using our own technology to get known compounds in gram to kilogram quantities. Using this technology [59], several novel DGs were purified in hundreds of milligrams and gram quantities [40]. This technology also provides an economic and convenient approach to the isolation and purification of larger quantities of potentially interesting natural products for confirmation of their bioactivities.

Two main approaches have been used for scaling-up the purification process of DGs: utilization of reverse phase (C-18) and silica gel stationary phases. Several sized columns were utilized for the isolation and purification of DGs: (1 × 25 cm, 5 µm), preparative (2.1 × 40 cm, 10 µm) and large-scale high-performance chromatography (7.5 × 50 cm, 10 µm) [59]. Reverse phase chromatography is perceived to have economic advantages over normal phase chromatography due to the usual practice of replacement of the normal phase adsorbent after one or at most a few uses whereas the reverse phase adsorbent can be used for hundreds of separations. The most used mobile phase for purifying DGs in reverse phase chromatography is acidified mixtures of acetonitrile: water in different ratios depending on the analytes. The solvent mixtures after processing the fractions are recovered (c.a 70% in acetonitrile as an aqueous azeotrope) and re-used in the next chromatography after adjusting the ratios based on the density of the mixtures. Thus, we can save solvents and make the purification process of these compounds at large scale more economic.

On the other hand, silica gel stationary phase for chromatography is perceived to not be reusable or of a very limited useful life. Consequently, less expensive poor-quality normal phase adsorbents are typically employed in preparative column packings. The poorer quality normal phase adsorbent is usually of irregular shaped particles and possesses a wide particle size distribution which together provides overall poor chromatographic performance for the packed bed. High quality normal phase adsorbents are available with spherical particles and narrow particle size distributions. As quality normal phase adsorbent costs about $5000 or more per kilogram and as the adsorbent is perceived not to be reusable, development of silica gel preparative chromatographic processes has largely been avoided or not considered. In our own experience a high-performance preparative silica gel column (10 µm, spherical silica gel) was packed in 2009 and hundreds of separations have been performed until now without re-packing. Appropriate column regeneration and re-equilibration to the next mobile phase composition increases the lifetime of the stationary phase and restores the performance qualities of the column [60].

With the technologies outlined above, silica gel chromatographic processes can be developed providing cost savings to users through better performance, higher capacity, easier product recovery, less costly solvent recovery, and less costly solvent disposal. Compound recovery from the organic solvent of the mobile phase used in silica gel chromatography is easier and less expensive than from the water containing mobile phase used in reverse phase chromatography. At production scale, the energy required to recycle the normal phase organic solvents is significantly less than that of reversed phase aqueous solvents. The waste disposal costs are reduced for the normal phase organic solvents because of their usually higher BTU content.

One of the most common mobile phases for separating non-glycosylated steviol and related aglycones using this technology are mixtures of *n*-heptane: wet acidified EtOAc and/or *n*-heptane: *wa*EtOAc: MeOH [40]. However, the detection of these compounds is generally set at 205 or 210 nm, below the UV absorbance cut off wavelength of ethyl acetate. An alternative mobile phase to solve this problem is the use of methyl tert-butyl ether (MTBE). This mobile phase was useful for the separation of isosteviol from a mixture of steviol and other isomers [40].

Obviously, normal phase separation of the glycosides requires a more polar mobile phase. Mixtures of EtOAc: MeOH: H_2_O with 0.1% AcOH or MTBE: MeOH: H_2_O with 0.1% AcOH in different ratios have been widely used in our experience with good separation of several DGs. Both organic mobile phases are easily recovered, and the solvent composition ratios could be adjusted by scouting by TLC for subsequent chromatography.

All methodologies have been useful to purify several steviol glycosides most of them in gram quantities and could be applied for any natural product research. In Table 2 are shown all the tetracyclic diterpene reported as natural compounds from *S. rebaudiana*.

## 4. Structure Elucidation for Diterpene Glycosides

The diversity of DGs has been considerably enhanced with the discovery of several new compounds in the last decade. Herein, we summarize all the DGs isolated from *S. rebaudiana* and reported as natural compounds from 1931 to 2021 (Table 2). The main structural differences have been found in the number of sugar units linked to the aglycone at positions C-13 and C-19 and the different arrangements described, thus some rebaudioside families have been described. Nevertheless, some other DGs with an aglycone core different from steviol have also been reported, and chemical structures are compiled in Figure 1.

The isolation and structure elucidation of minor DGs in quantities not only requires appropriate instrumentation and several chromatographic steps but also high-quality spectroscopic and spectrometric equipment. NMR analysis is the most extensively used technique to elucidate unambiguously natural compounds. However, as an aid for structure elucidation of new steviol glycosides some alternative methods have been reported in the literature and described herein.

### 4.1. Reversed-Phase High-Performance Liquid Chromatography

High-performance liquid chromatograph is a very common instrument in most of the laboratories today. Thus, a method based on using reversed-phase C18 column retention times of several previously characterized natural DGs and others with no sugar at position C-19 was described as an aid for structure elucidation of new and known DGs [26]. Specifically, the method is helpful to identify known and detect novel aglycone-C13 oligosaccharide moieties and give some hints about C-19 linkages. Elution order of several DGs and their aglycone-C13 oligosaccharide substituted with different sugar arrangements are summarized in Table 3.

Steviolmonoside (Stev-mono); Iso-steviolbioside (Iso-Stevbio); Rubusoside (Rub); Steviolbioside (Stev-bio), Stevioside (Stev); Iso-Steviolbioside (Iso-Stevbio); Iso-Stevioside (Iso-Stev); Rebaudioside G_1_ (Reb G_1_); Rebaudioside G (Reb G); Dulcoside A_1_ (Dulc A_1_); Dulcoside A (Dulc A); Rebaudioside B (Reb B); Rebaudioside A (Reb A); Iso-Rebaudioside A_1_ (Iso-Reb A_1_); Iso-Rebaudioside A (Iso-Reb A); Dulcoside B (Dulc B); Rebaudioside C (Reb C); Rebaudioside F_1_ (Reb F_1_); Rebaudioside F (Reb F); Rebaudioside L_1_ (Reb L_1_); Rebaudioside L (Reb L); Rebaudioside H (Reb H); Rebaudioside H_1_ (Reb H_1_); **a:** 13-[(2-*O*-β-d-glucopyranosyl-3-*O*-β-d-glucopyranosyl-β-d-glucopyranosyl) oxy]*ent*-hydroxyatis-16-en-19-oic acid -β-d-glucopyranosy ester; **b:** 13-[(2-*O*-*β*-D-xylopyranosyl-*β*-D-glucopyranosyl-)oxy]*ent*-kaur-16-en-19-oic acid *β*-D-glucopyranosyl ester; **c:** 13-[(2-*O*-β-d-glucopyranosyl-3-*O*-β-d-glucopyranosyl-β-d-glucopyranosyl) oxy]*ent*-hydroxyatis-16-en-19-oic acid; **d:** 13-[(2-*O*-6-deoxy-*β*-D-glucopyranosyl-3-*O*-*β*-D-glucopyranosyl-*β*-D-glucopyranosyl)oxy]*ent*-kaur-16-en-19-oic acid *β*-D-glucopyranosyl ester; **e:** 13-[(2-*O*-β-D-xylopyranosyl-β-D-glucopyranosyl-)oxy]kaur-16-en-19-oic acid; **f:** 13-[(2-*O*-6-deoxy-β-D-glucopyranosyl-3-*O*-β-D-glucopyranosyl-β-D-glucopyranosyl)oxy]kaur-16-en-19-oic acid.

The number of sugar components may be inferred by retention time ranges: *DGs* with retention times < 7 min (oligosaccharide with five to seven units), although rebaudioside E or similar isomers, with two monosaccharide units at C-13 and C-19, four glucose units with a 1-6 linkage in C-13 or four glucose units attached to a an *ent*-atisene core show retention times under 7 min, *DGs* with retention times between 7 to 11.5 min (with a di or trisaccharide at C-13 and a monosaccharide at C-19, although three glucose units at C-13 with a 1-6 linkage or linked to an *ent*-atisene core also elute in this range), *DGs* with retention times between 11.6- 12.5 min (a tetrasaccharide oligomer is attached at C-13 and no sugar at C-19) while rubusoside, the only steviol glycosides reported with one sugar unit at both C-13 and C-19, elutes at 12.5 min and *DGs* with retention times greater than 1*2.5 min* (a mono, di or trisaccharide at C-13 and a free carboxylic acid at C-19). However, to confirm the identity of highly substituted DGs, a normal phase chromatographic method should be run.

The method is based on comparing retention times of a pure natural DG and its saponification product with those reported in Table 3. If the retention time of a natural DG and its saponification product match with one of those reported in Table 3, most probably the DG has previously been identified. During the analyses of novel DGs few possibilities may be found: (1) DGs with a novel C-13 and known C-19 arrangement; (2) DGs with known C-13 and new C-19 arrangement; (3) DGs with unknown C-13 and C-19 arrangements or (4) DGs with known C-13 and C-19 moieties but with a unique combination that makes the DGs become an undescribed structure e.g., rebaudioside V [26].

For identifying the sugar component numbers and arrangement at the C-13 moiety, the C-19 portion is cleaved under alkaline hydrolysis and clean up following acidification. HPLC retention time of the aglycone-C13 oligosaccharide moiety is obtained using specific concentration and chromatographic conditions [26]. Comparing retention times with those reported in Table 3 will provide rapid information about the C-13 oligosaccharide arrangement or its novelty.

The strength of the alkaline hydrolysis is also important to get hints about the number of saccharide units and infer their positions of the C-19 oligomer. Mild alkaline hydrolysis over 30 min easily cleaves the monosaccharide linked at position C-19 (rebaudioside A, stevioside, rebaudioside F, etc.). Ready cleavage also occurs when less hindered oligomers are attached at position C-19 e.g., rebaudioside I (Glcβ(1-3)Glcβ-) and rebaudioside U (Araα(1-6)Glc-). However, the same easy cleavage with a disaccharide hexose/pentose (1-4) hexose is expected. We also believe that an easy cleavage could happen in a trisaccharide or a tetrasaccharide with position 2 of the sugar directly linked at C-19 unsubstituted [26]. DGs with hindered disaccharide Glcβ(1-2)Glcβ- at position C-19 (Rebaudiosides D, E, J) and also trisaccharides and tetrasaccharides (with position 2 of the sugar directly bonded to C-19 substituent) as rebaudiosides M and O, respectively, only cleave completely using strong alkaline conditions (reflux, 1 h).

### 4.2. High-Resolution Electrospray Ionization Tandem Mass Spectrometry

As glycosidic compounds, DGs give greater ionization efficiencies in negative ion mode. The dissociation pattern of DGs has been carefully described by ranging collision energies (CE) using high-resolution electrospray ionization tandem mass spectrometry (HRESIMS) [73,74]. The understanding of the HRESIMS dissociation pattern of DGs is of great importance since this allows the rapid detection and identification of known and novel DGs. Obviously, chemical structures of new DGs should be confirmed by NMR experiments.

*Stevia* DGs are a good model to study the sequence of cleavage, by tandem mass spectrometry, of each sugar monomer from the parent ions due to the variety of types of sugar monomers and their arrangement in the oligosaccharide.

#### 4.2.1. Cleavage of the C-19 Moiety

It is well-known that monosaccharides and oligomers attached at position C-19 (ester portion) is the first moiety cleaved from DGs parent ions. Only glycosides with glucose directly attached at C-19 have been found so far. Ranging of collision energies (CE) allows one to infer important structural information about the C-19 moiety: number of sugar units at C-19 and position of sugar linkages. DGs containing a monosaccharide at C-19 position cleave easily at CE of 10 eV supported with the presence of two major ions in the MS spectrum, the deprotonated molecule [M-H]^−^ and the major product ion [M-H-162]- Da. All the DGs containing a monosaccharide at position C-19 analyzed by HRESIMS showed the same trend e.g., rebaudiosides A, G, H, L and stevioside among others. In the same way, DGs containing less hindered disaccharides at position C-19 also cleave at low collision energy. Rebaudiosides I (Glc(1-3)Glc-) and U (Ara(1-6)Glc-) are a couple of DGs with a less hindered disaccharide attached at C-19. Both compounds show cleavage of the C-19 moiety at 10 eV but produce lower product ion intensities: rebaudioside I-steviol-C13^−^, 14.4% intensity and rebaudioside U-steviol-C13^−^, 54% intensity. Product ion intensities of these less hindered C-19 disaccharides should be studied carefully since most probably the position of the second monosaccharide could be also inferred.

However, DGs with two sugars at both positions, C-13 and C-19, show different product ion intensities of the steviol with the C-13 moiety [steviol-C13]^−^ at 10 eV. Rebaudiosides E and S, the only examples at hand, showed both the deprotonated molecule ion [M-H]^−^ and the product ion [steviol-C13]^−^. Nevertheless, significantly lower product ion intensities were observed: rebaudioside E, [M-H-324]^−^ Da with *m*/*z* = 641 Da and intensity of 5.9% due to the loss of two glucoses and rebaudioside S, [M-H-308]^−^ Da with *m*/*z* = 641 Da and intensity of 7.5% with loss of one glucose and one rhamnose.

On the other hand, DGs containing hindered disaccharides (Rebaudioside D, E, K, etc.) and longer oligomer chains (Rebaudioside s M, N, O, W, etc.) at position C-19 with a C-13 moiety of more than two sugar units do not cleave at low CE. Due to this highly hindered ester, 40 eV needed to be applied to cleave the C-19 portion and produce detectable [steviol-C13 glycoside]^−^ product ions. We also expect a relatively easy cleavage at low CE of the C-19 moiety in DGs with a tri and tetrasaccharide oligomer at C-19 with the C-2 of the glucose directly linked to C-19 unsubstituted.

#### 4.2.2. Cleavage of the C-13 Moiety

**Figure 5:**
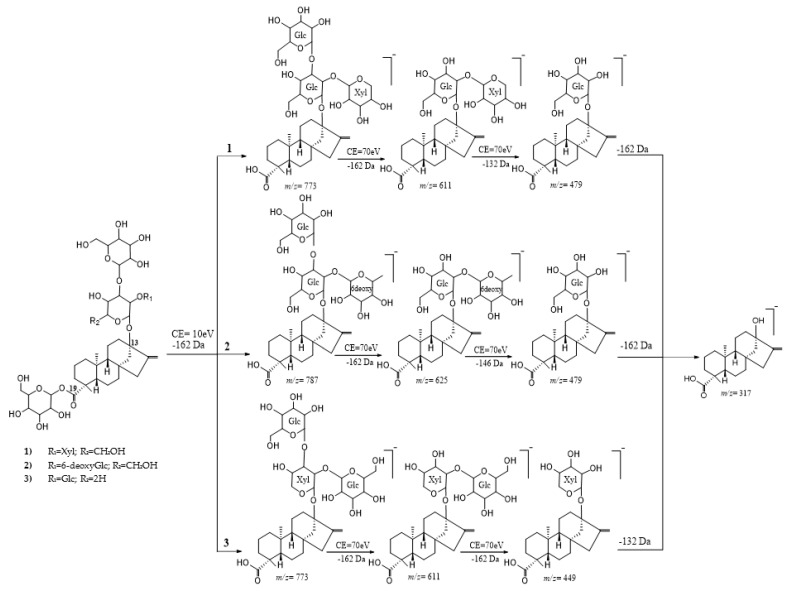
Fragmentation pattern of steviol glycosides.

In contrast to the C-19 moiety, a broad cleavage of the C-13 moieties in DGs occurs at 70 eV. In branched trisaccharides at C-13, sugars at C-3 cleave first, followed by sugars at C-2 and finally sugars directly linked at C-13. The dissociation pattern of three tetraglycosides with a glucose attached at C-19 and deoxyGlc*β*(1-2)[Glc*β*(1-3)]Glc*β*_1_- (13-[(2-*O*-6-deoxy-*β*-D-glucopyranosyl-3-*O*-*β*-D-glucopyranosyl*-β*-D-glucopyranosyl) oxy]*ent*-kaur-16-en-19-oic acid *β*-D-glucopyranosyl ester), Xyl*β*(1-2)[Glc*β*(1-3)]Glc*β*_1_- (rebaudioside F) and Glc*β*(1-2)[Glc*β*(1-3)]Xyl*β*_1_- (rebaudioside R) are three DG examples that allow the understanding of the sequential loss of each sugar unit from C-13 (Figure 5).

Rebaudiosides H, L, VIII, IX and IXd are the only reported examples of natural DGs with more than three monosaccharide units at C-13. Rebaudiosides L, VIII and series IX contain only glucoses which makes it difficult to understand the sequential cleavage of C-13 moieties. However, it was found that rebaudioside H [Glc(1-3)Rha*α*(1-2)[Glc*β*(1-3)]Glc*β*_1_-] showed a sequential loss of four sugar units at CE (70 eV) following the C-19 cleavage as follows: first two glucoses, *m*/*z* = 787.3729 Da and 625.3197 Da, followed by a rhamnose *m*/*z* = 479.2637 Da and a further glucose loss *m*/*z* = 317.2106 Da. Probably, the glucose attached at C-3 of the rhamnose is the first cleaved followed by glucose at C-3 of the glucose directly attached at C-13. DGs have been grouped into 11 groups by molecular weights or structure similarities for an ease of understanding of the different dissociation pattern of several DGs and isomers. MS data of several natural and prepared DGs have been recently reported [74].

### 4.3. Determination of the Absolute Configuration of the Monosaccharides.

A simple and rapid reversed-phase C18 high-performance and ultra-high-performance liquid chromatography methods have been developed to determine the absolute configuration of several monosaccharides linked to different kind aglycones [75,76]. This method is not only appropriate to DGs from *S. rebaudiana*, but it can also be applied for several classes of glycosides: flavonoid glycosides, alkaloid glycosides and triterpene glycosides [77,78] among others. The method is based on cleavage of all the monosaccharides linked to the aglycone under acid hydrolysis. Further liquid-liquid partitions with adequate organic solvent are needed to separate the monosaccharides from the aglycone. It is well-known in steviol glycosides that steviol is not the main aglycone recovered but rather a mixture of isosteviol, endo-steviol and steviol. Basically, two step reactions are needed to yield monosaccharide derivatives traceable by UV with enough difference in retention times between D and L monosaccharide enantiomers. The first step reaction is between the monosaccharides and L-cysteine methyl ester to yield the thiazolidine derivatives followed with further addition of phenylisothiocyanate to yield the thiocarbamoyl thiazolidine monosaccharide derivatives. Both step reactions are performed at 60–70 °C in pyridine. The identity and absolute configuration of the monosaccharide could be identified by comparison of the retention times of the derivatives with appropriate standards [75,76]. Thiohydantoin is a by-product yielded in the reaction which has earlier retention times in RP-C18 HPLC method than the thiocarbamoyl-thiazolidine sugar derivatives [75].

## 5. NMR Experiments

**Figure 6:**
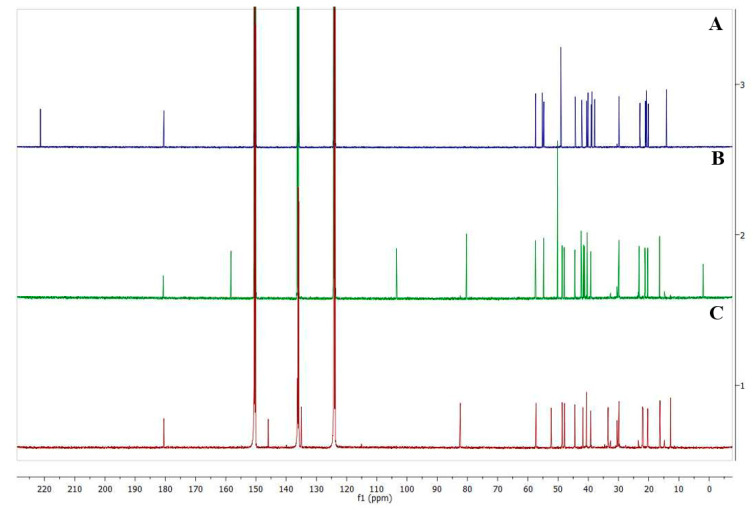
Comparison of the ^13^C-NMR of isosteviol (**A**), steviol (**B**) and endocyclic steviol isomer (**C**).

Several approaches have been discussed in previous sections, all of them provide important information for the structure elucidation of the DGs but still those approaches do not fully elucidate the structures of several DGs. The oligosaccharides at position C-19 and isomeric aglycones are not easily identified using previous methods. There is no doubt that NMR is the most powerful technique for the structure elucidation of any compound if an appropriate amount and purity are in hand. Steviol is the main aglycone in DGs from *S. rebaudiana*, although other natural and degradation cores have also been reported (Figure 1). As far as we know, only steviol, isosteviol, endo steviol and the aglycone with the hydrated double bond at 16,17 (Figure 1V) have been isolated as the aglycone forms. The ^13^C-NMR spectra for the aglycones of isosteviol, steviol and endocyclic steviol isomer are shown in Figure 6. The ^1^H and ^13^C NMR chemical shifts of the main aglycones found in *S. rebaudiana* or in DGS from leaves of this plant are listed (Table 4).

Herein, we discuss the key NMR chemical shifts to differentiate the aglycone cores from *S. rebaudiana*. Steviol has present an exocyclic double bond at position C-16 and the glycosylation sites at position C-13 and C-19 (Figure 1I). The double bond is characterized by a quaternary (158.3 ppm) and a methylene olefinic carbon resonance (103.5 ppm, ^13^C resonances); and proton resonances (5.48 and 5.04 ppm) while endo steviol, has an endocyclic double bond at position C-15 (135.0, ^13^C) and 5.24 ppm ^1^H) and C-16 (145.9 ppm ^13^C) with an additional methyl group at C-17 (12.7 ^13^C and 1.59 ppm ^1^H) (Figure 1IV). Isosteviol is easily differentiated from the other aglycones due to the presence of a ketone group (221.3 ppm ^13^C) at C-16 and an additional methyl group (29.9 ^13^C and 1.40 ppm ^1^H) (Figure 1III). Compounds 7 with a molecular weight of (332 Da) (Figure 1VI) and 8 with 334 Da (Figure 1VII) possess an endocyclic double bond like endo steviol. The slight structural differences are found at position C-17 where a remarkable difference in chemical shifts is observed, compound 7 has an -CHO (191.4 ^13^C and 9.61 ppm ^1^H) and 8 a -CH_2_OH group (59.2 ^13^C and 4.11; 4.29 ^1^H). Different from the other cores, compound 4 with a molecular weight of 336 Da (Figure 1V), does not present any double bond, the main difference is found in the groups linked at position C-16, -CH_3_ (22.2 ^13^C; 1.32 ppm ^1^H) and -OH (77.1 ppm ^1^H).

Compounds 2 (Figure 1VII) and 6 (Figure 1II) share very similar chemical shifts, although different structures have been assigned. Compound 6 was unambiguously elucidated using appropriate 1D and 2D NMR experiments and aglycone was defined as ***ent***-13(***S***)-hydroxyatisenoic acid core. If compared with steviol, the main differences were found in C-13 (77.8 ppm ^13^C), C-14 (37.7 ppm ^13^C), C-16 (146.7 ppm ^13^C) and C-17 (108.0 ppm ^13^C). Structure of compound 2 should be reanalyzed. Compound 9 shows similar chemical shifts that steviol aglycone with a main difference in the chemical shifts of H-15 and C-15 with 3.70 ppm and 80.8, respectively. The NMR chemical shifts of selected DGs with similar structures are compiled from Table 5, Table 6, Table 7 and Table 8. In Table 5 are shown the NMR data for DGs with three glucose units while in Table 6, DGs with four glucose units.

In Table 7 are presented DGs with four sugar units, one xylose and three glucoses with different arrangements whereas in Table 8, a few examples of DGs with four sugar units, one rhamnose or 6-deoxyglucose together with three glucose with different arrangements.

## 6. Conclusions

*Stevia rebaudiana* and its steviol glycosides have been widely studied, and in the continuous search for non-caloric sugar substitutes with improved taste, multiple new tetracyclic diterpene glycosides have been isolated from leaf of *S. rebaudiana* or have been prepared by chemical or enzymatic reactions from selected steviol glycosides. Herein, an updated list of natural DGs isolated from *S. rebaudiana* has been compiled, along with some chemically modified DGs. Some approaches for the rapid detection of new DG structures in fractions rich in DGs have also been presented. Thus, the fragmentation pattern for DGs by High-Resolution Electrospray Ionization Mass Spectrometry has been presented. The modification of DGs by a simple saponification reaction with further analysis of the products by Reverse-Phase High-Performance Liquid Chromatography for the detection of new moieties at position C-13 is another approach summarized in this review. Several HPLC methods with diverse stationary phases, but with a unique mobile phase used for the analysis of fractions rich of specific DGs were discussed. However, a major drawback in the search of novel structures in natural products, is the isolation of quantities that are insufficient to allow bioassays or in the case of DGs, conducting tasting assays to better understand the relationship structure-sweetness/bitterness of the DGs, and in turn followed by toxicological evaluation. Some strategies to save cost in the scale-up of the purification process were also shared. With the described strategies, several very minor DGs were purified in quantities of hundreds of milligrams to multiple grams. Currently, several DGs with different aglycone cores, numbers and types of sugar units, and arrangements have been well documented so far. Hence, the number of DG structures available is vast, although the reported tasting results are infrequent or at least not visible in the scientific literature. Further studies need to be pointed out for the tasting evaluation of the DGs already discovered. With the availability of this information, adequate strategies could be followed to overexpress specific groups of DGs in *S. rebaudiana* and/or to use appropriate blends of DGs with improved taste.

## Figures and Tables

**Table 1:** Compounds reported as degradation products from natural diterpene glycosides by alkaline or acid conditions. **a** 13-[(2-*O*-*β*-D-xylopyranosyl-*β*-D-glucopyranosyl-)oxy]*ent*-kaur-16-en-19-oic acid *β*-D-glucopyranosyl ester; **b** 13-[(2-*O*-6-deoxy-*β*-D-glucopyranosyl-3-*O*-*β*-D-glucopyranosyl-*β*-D-glucopyranosyl)oxy]*ent*-kaur-16-en-19-oic acid *β*-D-glucopyranosyl ester.

DGs from Alkaline Hydrolysis							
Common Name	Oligosaccharide Moieties		AS	Chemical Formula	Accurate MW	Starting Material	Ref
	C-13	C-19					
-	Xylβ(1-2)Glcβ_1_-	-	**I**	C_31_H_48_O_12_	612.3146	a	[26]
Dulcoside A_1_	Rhaα(1-2)Glcβ_1_-	-	**I**	C_32_H_50_O_12_	626.3303	Dulcoside A	[26]
Rebaudioside G_1_	Glcβ(1-3)Glcβ_1_-	-	**I**	C_32_H_50_O_13_	642.3252	Rebaudioside G	[26]
Rebaudioside F_1_	Xylβ(1-2)[Glcβ(1-3)]Glcβ_1_-	-	**I**	C_37_H_58_O_17_	774.3675	Rebaudioside F	[11,26]
Rebaudioside R_1_	Glc(1-2)[Glcβ(1-3)]Xylβ1-	-	**I**	C_37_H_58_O_17_	774.3675	Rebaudioside R	[26]
Rebaudioside Z_1_	Glcβ(1-6)[Glcβ(1-2)]Glcβ_1_-	-	**I**	C_38_H_60_O_18_	804.3781	Rebaudioside Z	[31]
-	Glcβ(1-2)[Glcβ(1-3)]Glcβ_1_-		**II**	C_38_H_60_O_18_	804.3781	-	[31]
-	6-deoxyGlcβ(1-2)[Glcβ(1-3)]Glcβ_1_-	-	**I**	C_38_H_60_O_17_	788.3831	**b**	[26]
Rebaudioside H_1_	Glcβ(1-6)Glcβ(1-3)[Glcβ(1-3)]Glcβ_1_-	-	**I**	C_44_H_70_O_23_	966.4309	Rebaudioside H	[26]
Rebaudioside L_1_	Glcβ(1-3)Rhaα(1-2)[Glcβ(1-3)]Glcβ_1_-	-	**I**	C_44_H_70_O_23_	966.4309	Rebaudioside L	[26]
**DGs from acid hydrolysis**							
Isosteviol	-	-	**III**	C_20_H_30_O_3_	318.2195	Rebaudioside A/Stevioside	[34,38]
*Endo*-steviol	-	-	**IV**	C_20_H_30_O_3_	318.2195	Rebaudioside A/Stevioside	[34,38]
*Endo*-steviolmonoside	Glcβ_1_-	-	**IV**	C_26_H_40_O_8_	480.2724	Rebaudioside A/Rubusoside	[38,39]
*Endo*-rebaudioside G_1_	Glcβ(1-3)Glcβ_1_-	-	**IV**	C_32_H_50_O_13_	642.3252	Rebaudioside A	[38]
*Endo*-steviolbioside	Glcβ(1-2)Glcβ_1_-	-	**IV**	C_32_H_50_O_13_	642.3252	Rebaudioside A	[38]
*Endo*-rubusoside	Glcβ_1_-	Glcβ_1_-	**IV**	C_32_H_50_O_13_	642.3252	Rebaudioside A/Rubusoside	[38,39]
*Iso-stevioside/Endo*-stevioside	Glcβ(1-2)Glcβ_1_-	Glcβ_1_-	**IV**	C_38_H_60_O_18_	804.3781	Rebaudioside A	[38,41]
*Iso-rebaudioside B/Endo*-rebaudioside B	Glcβ(1-2)[Glcβ(1-3)]-Glcβ_1_-	-	**IV**	C_38_H_60_O_18_	804.3781	Rebaudioside A	[38,42]
-	-	Glcβ(1-2)[Glcβ(1-3)]Glcβ_1_-	**III**	C_38_H_60_O_18_	804.3781	Rebaudioside M	[35]
*Iso-rebaudioside AEndo*-rebaudioside A	Glcβ(1-2)[Glcβ(1-3)]Glcβ_1_-	Glcβ_1_-	**IV**	C_44_H_70_O_23_	966.4309	Rebaudioside A	[38]
-	Glcβ(1-2)[Glcβ(1-3)]Glcβ_1_-	Glcβ(1-2)[Glcβ(1-3)]Glcβ_1_-	**IV**	C_56_H_90_O_33_	1290.5366	Rebaudioside M	[35]
-	Glcβ(1-2)[Glcβ(1-3)]Glcβ_1_-	Glcβ(1-2)[Glcβ(1-3)]Glcβ_1_-	**V**	C_56_H_92_O_34_	1308.5472	Rebaudioside M	[35]

**Table 2:** Diterpene glycosides purified from *Stevia rebaudiana*.

Common Name	Oligosaccharide Moieties		AS	Chemical Formula	MW	Ref
	C-13	C-19				
Steviol	H	H	**I**	C_20_H_30_O_3_	318.2195	[61]
Steviol monoacetate	H	COCH_3_	**I**	C_22_H_32_O_4_	360.2192	[62]
Steviolmonoside	Glcβ_1_-	H	**I**	C_26_H_40_O_8_	480.2724	[16]
Steviol-19-O-β-D glucoside	H	Glcβ_1_-	**I**	C_26_H_40_O_8_	480.2724	[63]
Rubusoside	Glcβ_1_-	Glcβ_1_-	**I**	C_32_H_50_O_13_	642.3252	[16]
**Oxidized core family**						
Isosteviol-19-*O*-β-glucoside	-	Glcβ_1_-	**III**	C_26_H_40_O_8_	480.2724	[42]
-	Glcβ(1-2)Glcβ_1_-	Glcβ_1_-	**VI**	C_38_H_58_O_19_	818.3573	[37]
-	Glcβ(1-2)Glcβ_1_-	Glcβ_1_-	**VII**	C_38_H_60_O_19_	820.3728	[37]
-	Glcβ(1-2)[Glcβ(1-3)]Glcβ_1_-	Glcβ_1_-	**VII**	C_44_H_70_O_24_	982.4258	[64]
-	Glcβ(1-2)[Glcβ(1-3)]Glcβ_1_-	Glcβ_1_-	**IV**	C_44_H_72_O_24_	984.4415	[41]
**Rebaudioside A family**						
Steviolbioside	Glcβ(1-2)Glcβ_1_-	H	**I**	C_32_H_50_O_13_	642.3252	[7]
-	Glcβ_1_-	Glcβ(1-2)Glcβ_1_-	**I**	C_38_H_60_O_18_	804.3781	[22]
Rebaudioside KA	Glcβ(1-2)Glcβ_1_-	Glcβ_1_-	**VIII**	C_38_H_60_O_18_	804.3781	[22]
Stevioside	Glcβ(1-2)Glcβ_1_-	Glcβ_1_-	**I**	C_38_H_60_O_18_	804.3781	[6]
Rebaudioside B	Glcβ(1-2)[Glcβ(1-3)]Glcβ_1_-	H	**I**	C_38_H_60_O_18_	804.3781	[7]
Rebaudioside E	Glcβ(1-2)Glcβ_1_-	Glcβ(1-2)Glcβ_1_-	**I**	C_44_H_70_O_23_	966.4309	[9]
Rebaudioside A	Glcβ(1-2)[Glcβ(1-3)]Glcβ_1_-	Glcβ_1_-	**I**	C_44_H_70_O_23_	966.4309	[7]
-	Glcβ(1-6)Glcβ(1-2)Glcβ_1_-	Glcβ_1_-	**I**	C_44_H_70_O_23_	966.4309	[65]
Rebaudioside Y	Glcβ(1-2)Glcβ_1_-	Glcβ(1-6)Glcβ_1_-	**I**	C_44_H_70_O_23_	966.4309	[58]
Rebaudioside Z	Glcβ(1-6)[Glcβ(1-2)]Glcβ_1_-	Glcβ_1_-	**I**	C_44_H_70_O_23_	966.4309	[31]
Rebaudioside D	Glcβ(1-2)[Glcβ(1-3)]Glcβ_1_-	Glcβ(1-2)Glcβ_1_-	**I**	C_50_H_80_O_28_	1128.4838	[9]
Rebaudioside I	Glcβ(1-2)[Glcβ(1-3)]Glcβ_1_-	Glcβ(1-3)Glcβ_1_-	**I**	C_50_H_80_O_28_	1128.4838	[16]
Rebaudioside M	Glcβ(1-2)[Glcβ(1-3)]Glcβ_1_-	Glcβ(1-2)[Glcβ(1-3)]Glcβ_1_-	**I**	C_56_H_90_O_33_	1128.4838	[16]
Rebaudioside L	Glcβ(1-6)Glcβ(1-2)[Glcβ(1-3)]Glcβ_1_-	Glcβ_1_-	**I**	C_50_H_80_O_28_	1128.4838	[16]
-	Glcβ(1-6)[Glcβ(1-3)]Glcβ_1_-	Glcβ(1-2)Glcβ_1_-	**I**	C_50_H_80_O_28_	1128.4838	[41]
-	Glcβ(1-2)Glcβ_1_-	Glcβ(1-6)[Glcβ(1-2)Glcβ_1_-	**I**	C_50_H_80_O_28_	1128.4838	[66]
15α-hydroxy-Reb M	Glcβ(1-2)[Glcβ(1-3)]Glcβ_1_-	Glcβ(1-2)[Glcβ(1-3)]Glcβ_1_-	**IX**	C_56_H_90_O_34_	1306.5313	[30]
**Rebaudioside VIII and IX families**						
Rebaudioside VIII	Glcβ(1-2)[Glcβ(1-3)]-Glcα(1-6)-Glcβ(1-2)[Glcβ(1-3)]Glcβ_1_-	Glcβ(1-2)Glcβ_1_-	**I**	C_68_H_110_O_43_	1614.6420	[29]
Rebaudioside IX	Glcβ(1-2)[Glcβ(1-3)]-Glcα(1-6)-Glcβ(1-2)[Glcβ(1-3)]Glcβ_1_-	Glcβ(1-2)[Glcβ(1-3)]Glcβ_1_-	**I**	C_74_H_119_O_48_	1776.6949	[27]
Rebaudioside IXa	Glcβ(1-2)[Glcβ(1-3)]Glcβ_1_-	Glcβ(1-2)[Glcβ(1-3)]Glcβ(1-2)[Glcβ(1-3)]-Glcα(1-6)-Glcβ_1_-	**I**	C_74_H_119_O_48_	1776.6949	[28]
Rebaudioside IXb	Glcβ(1-2)[Glcβ(1-3)]Glcβ_1_-	Glcβ(1-2)[Glcβ(1-3)]-Glcα(1-6)-Glcβ(1-2)[Glcβ(1-3)]Glcβ_1_-	**I**	C_74_H_119_O_48_	1776.6949	[28]
Rebaudioside IXc	Glcβ(1-2)[Glcβ(1-3)]Glcβ_1_-	Glcβ(1-2)[Glcβ(1-2)[Glcβ(1-3)]-Glcα(1-3)-Glcβ(1-3)]Glcβ_1_-	**I**	C_74_H_119_O_48_	1776.6949	[28]
Rebaudioside IXd	Glcβ(1-2)[Glcβ(1-3)]Glcβ(1-2)[Glcβ(1-3)]-Glcα(1-6)-Glcβ_1_-	Glcβ(1-2)[Glcβ(1-3)]Glcβ_1_-	**I**	C_74_H_119_O_48_	1776.6949	[29]
**Rebaudioside C family**						
Dulcoside A	Rhaα(1-2)Glcβ_1_-	Glcβ_1_-	**I**	C_38_H_60_O_17_	788.3831	[8]
Dulcoside B	Rhaα(1-2)[Glcβ(1-3)]Glcβ_1_-	H	**I**	C_38_H_60_O_17_	788.3831	[16]
Rebaudioside C/Dulcoside B	Rhaα(1-2)[Glcβ(1-3)]Glcβ_1_-	Glcβ_1_-	**I**	C_44_H_70_O_22_	950.4356	[8,10]
Rebaudioside S	Glcα(1-2)Glcβ_1_-	Rhaα(1-2)Glcβ_1_-	**I**	C_44_H_70_O_22_	950.4356	[23]
-	Glcβ(1-2)Glcβ_1_-	Rhaα(1-2)Glcβ_1_-	**I**	C_44_H_70_O_22_	950.4358	[25]
Rebaudioside H	Glcβ(1-3)Rhaα(1-2)[Glcβ(1-3)]Glcβ_1_-	Glcβ_1_-	**I**	C_50_H_80_O_27_	1112.4888	
Rebaudioside K	Rhaα(1-2)[Glcβ(1-3)]Glcβ_1_-	Glcβ(1-2)Glcβ_1_-	**I**	C_50_H_80_O_27_	1112.4888	[16]
Rebaudioside J	Glcβ(1-2)[Glcβ(1-3)]Glcβ_1_-	Rhaα(1-2)Glcβ_1_-	**I**	C_50_H_80_O_27_	1112.4888	[16]
**Rebaudioside C family**						
-	Rhaα(1-2)[Glcβ(1-3)]Glcβ_1_-	Glcβ(1-6)Glcβ_1_-	**I**	C_50_H_80_O_27_	1112.4887	[25]
Rebaudioside N	Glcβ(1-2)[Glcβ(1-3)]Glcβ_1_-	Rhaα(1-2)[Glcβ(1-3)]Glcβ_1_-	**I**	C_56_H_90_O_32_	1274.5417	[16]
Rebaudioside O	Glcβ(1-2)[Glcβ(1-3)]Glcβ_1_-	Glcβ(1-3)Rhaα(1-2)[Glcβ(1-3)]Glcβ_1_-	**I**	C_62_H_100_O_37_	1436.5945	[16]
**Glcβ(1-3)Glcβ_1_- family**						
Rebaudioside G	Glcβ(1-3)Glcβ_1_-	Glcβ_1_-	**I**	C_38_H_60_O_18_	788.3831	[16]
**Rebaudioside F family**						
-	Xylβ(1-2)Glcβ_1_-	Glcβ_1_-	**I**	C_37_H_58_O_17_	774.3675	[67]
Rebaudioside F	Xylβ(1-2)[Glcβ(1-3)]Glcβ_1_-	Glcβ_1_-	**I**	C_43_H_68_O_22_	936.4203	[11]
-	Glcβ(1-2)[Xylβ(1-3)]Glcβ_1_-	Glcβ_1_-	**I**	C_43_H_68_O_22_	936.4203	[67]
-	Glcβ(1-2)Glcβ_1_-	Xylβ(1-6)Glcβ_1_-	**I**	C_43_H_68_O_22_	936.4203	[37]
Rebaudioside R	Glcβ(1-2)[Glcβ(1-3)]Xylβ_1_-	Glcβ_1_-	**I**	C_43_H_68_O_22_	936.4203	[23]
Rebaudioside V	Xylβ(1-2)[Glcβ(1-3)]Glcβ_1_-	Glcβ(1-2)Glcβ_1_-	**I**	C_49_H_78_O_27_	1098.4730	[26]
-	Glcβ(1-2)[Glcβ(1-3)]Glcβ_1_-	Xylβ(1-2)Glcβ_1_-	**I**	C_49_H_78_O_27_	1098.4730	[25]
-	Glcβ(1-2)Glcβ_1_-	Xylβ(1-2)[Glcβ(1-4)]Glcβ_1_-	**I**	C_49_H_78_O_27_	1098.4730	[25]
Rebaudioside T	Glcβ(1-2)[Glcβ(1-3)]Glcβ_1_-	Xylβ(1-2)[Glcβ(1-3)]Glcβ_1_-	**I**	C_55_H_88_O_32_	1260.5260	[24,68]
-	Glcβ(1-2)[Xylβ (1-3)]Glcβ_1_-	Glcβ(1-2)[Glcβ(1-3)]Glcβ_1_-	**I**	C_55_H_88_O_32_	1260.5260	[25]
**Fruβ family**						
-	Glcβ(1-2)[Fruβ(1-3)]Glcβ_1_-	Glcβ_1_-	**I**	C_44_H_70_O_23_	966.4309	[65]
**Glcα family**						
-	Glcβ(1-2)Glcβ_1_-	Glcα(1-2)[Glcα(1-4)]-Glcβ_1_-	**I**	C_50_H_80_O_28_	1128.4838	[69]
-	Glcα(1-3)Glcβ(1-2)[Glcβ(1-3)]Glcβ_1_-	Glcβ_1_-	**I**	C_50_H_80_O_28_	1128.4838	[70]
-	Glcα(1-4)Glcβ(1-3)[Glcβ(1-2)]Glcβ_1_-	Glcβ_1_-	**I**	C_50_H_80_O_28_	1128.4838	[70]
		6-deoxyGlcβ family				
-	6-deoxyGlcβ(1-2)Glcβ_1_-	Glcβ_1_-	**I**	C_38_H_60_O_17_	788.3831	[71]
-	6-deoxyGlcβ(1-2)[Glcβ(1-3)]Glcβ_1_-	Glcβ_1_-	**I**	C_44_H_70_O_22_	950.4356	[71]
-	Glcβ(1-2)[Glcβ(1-3)]Glcβ_1_-	6-deoxyGlcβ_1_-	**I**	C_44_H_70_O_22_	950.4356	[72]
**Rebaudioside W family (three different sugar moieties)**						
Rebaudioside W	Xylβ(1-2)[Glcβ(1-3)]-Glcβ_1_-	Rhaα(1-2)[Glcβ(1-3)]Glcβ_1_-	**I**	C_55_H_88_O_31_	1244.5311	[26]
**Rebaudioside U Araα family**						
Rebaudioside U/6	Glcβ(1-2)[Glcβ(1-3)]Glcβ_1_-	Araα(1-6)Glcβ_1_-	**I**	C_49_H_78_O_27_	1098.4730	[24,25]
-	Glcβ(1-2)[Glcβ(1-3)]Glcβ_1_-	Araα(1-6)[Glcβ(1-2)]Glcβ_1_-	**I**	C_55_H_88_O_32_	1260.5258	[25]
***Ent*-atisene-core family**						
-	Glcβ(1-2)[Glcβ(1-3)]Glcβ_1_-	Glcβ_1_-	**II**	C_44_H_70_O_23_	966.4309	[31]
Stevatisene J	Glcβ(1-2)[Glcβ(1-3)]Glcβ_1_-	Rhaα(1-2)Glcβ_1_-	**II**	C_50_H_80_O_27_	1112.4888	[32]
Stevatisene K	Rhaα(1-2)[Glcβ(1-3)]Glcβ_1_-	Glcβ(1-2)Glcβ_1_-	**II**	C_50_H_80_O_27_	1112.4888	[32]
Stevatisene T	Glcβ(1-2)[Glcβ(1-3)]Glcβ_1_-	Xylβ(1-2)[Glcβ(1-3)]Glcβ_1_-	**II**	C_55_H_88_O_32_	1260.5260	[32]
Stevatisene N	Glcβ(1-2)[Glcβ(1-3)]Glcβ_1_-	Rhaα(1-2)[Glcβ(1-3)]Glcβ_1_-	**II**	C_56_H_90_O_32_	1274.5417	[32]
Stevatisene O	Glcβ(1-2)[Glcβ(1-3)]Glcβ_1_-	Glcβ(1-3)Rhaα(1-2)[Glcβ(1-3)]Glcβ_1_-	**II**	C_62_H_100_O_37_	1436.5945	[32]

**Table 3:** Diterpene glycosides organized by increasing retention times in an RP-C18 HPLC method.

DG	R_T_ (min)	Δ_RT_ (min)	Oligosaccharide Positions	
			C-13	C-19
Reb O	2.92	0.16	Glcβ(1-2)[Glcβ(1-3)]Glcβ_1_-	Glcβ(1-3) Rhaα(1-2)[Glcβ(1-3)]-Glcβ_1_-
Reb N	3.08	-	Glcβ(1-2)[Glcβ(1-3)]Glcβ_1_-	Rhaα(1-2) [Glcβ(1-3)]-Glcβ_1_-
Reb E	3.08	0.18	Glcβ_1_(1-2)Glcβ_1_-	Glcβ_1_(1-2)Glcβ_1_ -
Reb D	3.26	0.23	Glcβ(1-2)[Glcβ(1-3)]Glcβ_1_-	Glcβ_1_(1-2)Glcβ_1_ -
Reb J	3.49	0.07	Glcβ(1-2)[Glcβ(1-3)]Glcβ_1_-	Rhaα (1-2)Glcβ_1_-
Reb W	3.56	0.20	Xylβ(1-2)[Glcβ(1-3)]Glcβ_1_-	Rhaα(1-2) [Glcβ(1-3)]-Glcβ_1_-
Reb M	3.76	0.04	Glcβ(1-2)[Glcβ(1-3)]Glcβ_1_-	Glcβ(1-2)[Glcβ(1-3)]-Glcβ_1_-
Reb Y	3.80	0.09	Glcβ(1-2)Glcβ_1_-	Glcβ_1_(1-6)Glcβ_1_-
Reb V	3.89	0.05	Xylβ(1-2)[Glcβ(1-3)]Glcβ_1_-	Glcβ_1_(1-2)Glcβ_1_ -
Reb Z	3.94	0.24	Glcβ(1-2)[Glcβ(1-6)]-Glcβ1-	Glcβ_1_-
Reb U	4.18	0.17	Glcβ(1-2)[Glcβ(1-3)]Glcβ_1_-	Araα(1-6)-Glcβ_1_-
a	4.35	0.50	Glcβ(1-2)[Glcβ(1-3)]Glcβ_1_-	Glcβ_1_-
Reb T	4.85	0.61	Glcβ(1-2)[Glcβ(1-3)]Glcβ_1_-	Xylβ(1-2)[Glcβ(1-3)]-Glcβ_1_-
Reb H	5.46	0.18	Glcβ(1-3)Rhaα(1-2)[Glcβ(1-3)]Glcβ_1_-	Glcβ_1_-
Reb L	5.64	0.71	Glcβ(1-6)Glcβ(1-3)[Glcβ(1-3)]Glcβ_1_-	Glcβ_1_-
Reb I	6.35	0.76	Glcβ(1-2)[Glcβ(1-3)]Glcβ_1_-	Glcβ_1_(1-3)Glcβ_1_-
Reb A	7.11	0.42	Glcβ(1-2)[Glcβ(1-3)]Glcβ_1_-	Glcβ_1_-
Stev	7.53	0.38	Glcβ(1-2)Glcβ_1_-	Glcβ_1_-
Iso-Reb A	7.91	0.65	Glcβ(1-2)[Glcβ(1-3)]Glcβ_1_-	Glcβ_1_-
Iso-Stev	8.56	0.52	Glcβ(1-2)Glcβ_1_-	Glcβ_1_-
Reb F	9.08	0.24	Xylβ(1-2)[Glcβ(1-3)]Glcβ_1_-	Glcβ_1_-
Reb Z_1_	9.32	0.47	Glcβ(1-2)[Glcβ(1-6)]Glcβ1-	H-
Reb C	9.79	0.13	Rhaα(1-2)[Glcβ(1-3)]Glcβ_1_-	Glcβ_1_-
Reb R	9.92	0.34	Glc(1-2)[Glcβ(1-3)]Xylβ1-	Glcβ_1_-
b	10.26	0.16	Xylβ(1-2)Glcβ_1_-	Glcβ_1_-
Dulc A	10.42	0.08	Rhaα(1-2)Glcβ_1_-	Glcβ_1_-
c	10.50	0.69	Glcβ(1-2)[Glcβ(1-3)]Glcβ_1_-	-
Reb G	11.19	0.26	Glcβ(1-3)Glcβ_1_-	Glcβ_1_-
d	11.45	0.56	6-deoxyGlcβ(1-2)[Glcβ(1-3)]Glcβ_1_-	Glcβ_1_-
Reb L_1_	12.01	0.28	Glcβ(1-3) Rhaα(1-2)[Glcβ(1-3)]Glcβ_1_-	H-
Reb H_1_	12.29	0.23	Glcβ(1-6) Glcβ(1-3)[Glcβ(1-3)]Glcβ_1_-	H-
Rub	12.52	1.80	Glcβ_1_-	Glcβ_1_-
Reb B	14.32	0.12	Glcβ(1-2)[Glcβ(1-3)]Glcβ_1_-	H-
Iso-Reb B	14.44	0.44	Glcβ(1-2)[Glcβ(1-3)]Glcβ_1_-	H-
Stev-bio	14.88	0.29	Glcβ(1-2)Glcβ_1_-	H-
Iso-Stevbio	15.17	0.15	Glcβ(1-2)Glcβ_1_-	H-
Dulc B	15.31	0.52	Rhaα(1-2)[Glcβ(1-3)]Glcβ_1_-	H-
Reb F_1_	15.83	0.61	Xylβ(1-2)[Glcβ(1-3)]Glcβ_1_-	H-
Dulc A_1_	16.45	0.12	Rhaα(1-2)Glcβ_1_-	H-
Reb R_1_	16.57	0.70	Glc(1-2)[Glcβ(1-3)]Xylβ1-	H-
e	17.26	0.02	Xylβ(1-2)Glcβ_1_-	H-
Reb G_1_	17.28	0.1	Glcβ(1-3)Glcβ_1_-	H-
f	17.36	2.19	6-deoxyGlcβ(1-2)[Glcβ(1-3)]Glcβ_1_-	H-
Stev-mono	19.55		Glcβ_1_-	H-

**Table 4:** ^1^H and ^13^C chemical shifts of the compounds **1**–**9**.

Position	1 ^(a)^		2 ^(b)^		3 ^(a)^		4 ^(a)^		5 ^(a)^	
	δ_H_	δ_C_	δ_H_	δ_C_	δ_H_	δ_C_	δ_C_		δ_H_	δ_C_
1	0.87; 1.87	41.5	0.79	40.2	0.92; 1.92	41.8	0.78; 1.75	40.3	0.88; 1.67	40.6
2	2.28; 1.52	20.3	2.13; 1.38	19.7	2.32; 1.56	20.4	1.34; 2.23	19.3	2.23; 1.46	20.1
3	2.05; 1.87	41.3	2.36	39.1	2.50; 1.11	39.2	1.00; 2.32	38.4	1.61; 1.33	37.9
4	-	44.4	-	44.6	-	44.4	-	43.8	-	44.3
5	1.11	57.5	1.03	57.9	1.14	57.3	1.04	57.1	1.14	57.4
6	2.07; 2.21	23.2	2.41; 1.84	21.1	2.23; 2.07	22.1	2.11; 2.43	23.1	2.04; 2.04	22.9
7	1.55; 1.45	42.4	2.78	39.6	1.90; 1.79	33.4	1.37; 1.88	42.8	1.66; 1.66	42.1
8	-	42.3	-	34.9	-	48.7	-	*NA*	-	49.1
9	1.02	54.8	1.01	51.7	0.97	47.9	0.84	54.8	1.14	55.2
10	-	40.3	-	44.6	-	40.6	-	*NA*	-	38.8
11	1.79; 1.55	21.3	1.59	27.3	1.72; 1.72	21.9	1.52; 1.71	19.8	1.60; 1.20	21.1
12	2.48; 1.09	39.2	4.11	78.4	1.75; 1.60	40.6	1.85; 2.67	31.6	2.45; 1.09	39.0
13	-	80.3	2.94	42.0	-	82.4	-	87.6	-	40.1
14	2.35; 1.58	48.7	1.42; 1.05	39.9	2.47; 1.77	52.3	2.44; 2.58	40.3	1.49; 1.38	54.7
15	2.25; 2.22	48.0	2.23; 1.91	49.0	5.24	135.0	1.41; 1.83	54.3	2.71; 2.66	49.1
16	-	158.3	-	148.4	-	145.9	-	77.1	-	221.3
17	5.48; 5.04	103.5	5.24; 4.90	109.5	1.89	12.7	1.32	22.2	1.04	20.7
18	1.28	29.9	1.26	29.1	1.38	29.8	1.28	27.7	1.40	29.9
19	-	180.7	-	177.4	-	180.6	-	176.9	-	180.7
20	1.20	16.4	1.04	13.3	1.24	16.3	1.31	16.0	0.98	14.1
**Position**	**6 ^(b)^**		**7 ^(b)^**		**8 ^(b)^**		**9 ^(b)^**			
	**δ_H_**	**δ_H_**	**δ_H_**	**δ_C_**	**δ_H_**	**δ_C_**	**δ_C_**	**δ_H_**		
1	0.92; 1.59	39.5	0.89; 1.88	41.7	0.86; 1.86	41.8	0.86; 1.89	40.9		
2	1.92; 1.40	18.4	1.42; 1.95	19.7	1.41; 1.96	19.8	1.45; 2.15	20.1		
3	2.20; 1.09	37.7	1.07; 2.17	38.6	1.06; 2.14	38.8	1.09; 2.06	39.0		
4	1.12	43.7	-	44.3	-	44.7	-	45.1		
5	1.78; 2.05	57.1	1.18	57.6	1.12	58.0	1.07	58.2		
6	1.57; 1.16	19.9	1.88; 2.05	21.1	1.83; 2.00	21.5	1.72; 2.08	23.6		
7	1.09	38.8	1.66; 1.75	39.0	1.55; 1.65	40.2	1.33; 1.68	36.7		
8	1.63; 1.45	33.9	-	50.6	-	49.5	-	46.4		
9	2.51	51.0	1.00	46.1	0.90	47.7	0.94	54.3		
10	4.04	38.1	-	40.5	-	40.1	-	40.9		
11	1.15; 2.51	26.3	1.77; 1.82	20.6	1.63; 1.72	21.8	1.40; 1.76	20.0		
12	1.89; 2.11	41.0	1.74; 1.87	31.4	1.69; 1.81	30.4	1.45; 2.16	38.7		
13	4.70; 4.84	77.8	-	88.5	-	90.2	-	88.7		
14	1.24	37.7	1.95; 2.32	47.7	1.73; 2.32	49.6	1.66; 2.16	40.8		
15	0.89	47.4	6.57	158.0	5.36	136.6	3.70	80.8		
16	0.92; 1.59	146.7	-	147.7	-	146.9	-	157.2		
17	1.92; 1.40	108.0	9.61	191.4	4.11; 4.29	59.2	5.31; 5.54	110.2		
18	2.20; 1.09	27.5	-	178.0	-	178.4	1.25	28.5		
19	1.12	176.8	1.22	28.5	1.21	28.6	-	178.8		
20	1.78; 2.05	11.9	1.02	16.0	1.00	15.8	0.97	17.3		

^(a)^ NMR spectra were recorded in Pyr-*d*_5_. ^(b)^ MeOH-*d*_4_, **1**: steviol; **2:** C-12 aglycone; **3:** endo steviol isomer; **4:** aglycone with a CH_3_ and OH groups linked at C-16; **5:** isosteviol; **6:** *ent*-atisene core from 13-[(2-O-β-D-glucopyranosyl-3-O-β-D-glucopyranosyl-β-d-glucopyranosyl) oxy] ent-hydroxyatis-16-en-19-oic acid -β-D-glucopyranosy ester; **7:** aglycone with a CHO group linked at C-16; **8:** aglycone with a CH_2_OH group linked at C-16; **9:** aglycone from 15α-hydroxy-rebaudioside M. Chemical structures of the aglycone cores are presented in Figure 1: steviol (I); *ent*-atisene (II); isosteviol (III); endo steviol (IV); CH_3_ and OH at C-16 (V); CHO group at C-16 (VI); CH_2_OH at C-16 (VII); C-12 linkage (VIII); 15-α-hydroxy-rebaudioside M (IX).

**Table 5:** ^1^H and ^13^C chemical shifts of the compounds **10**–**13**.

Moiety	Position	10 ^(a)^		11 ^(b)^		12 ^(b)^		13 ^(a)^	
		δ_H_	δ_C_	δ_H_	δ_C_	δ_H_	δ_C_	δ_H_	δ_C_
Aglycone	1	1.71; 0.73	40.9	0.76; 1.75	40.8	0.79	40.2	*NA*	40.7
	2	2.21; 1.42	19.5	1.70; 2.17	20.2	1.38; 2.13	19.7	*NA*	19.2
	3	2.33; 1.02	38.5	1.82; 2.14	38.9	2.36	39.1	*NA*	38.1
	4	--	44.1	--	44.5	--	44.6	*NA*	43.9
	5	1.05; 2.45	57.5	0.99	57.6	1.03	57.9	*NA*	57.3
	6	2.45; 1.90	22.3	1.91; 2.20	22.2	1.84; 2.41	21.1	*NA*	22.0
	7	1.26	41.8	1.31; 1.51	41.9	2.78	39.6	*NA*	41.5
	8	--	42.7	--	42.2	--	34.9	*NA*	42.5
	9	0.86	54.0	0.93	54.2	1.01	51.7	*NA*	53.8
	10	--	39.9	--	39.8	--	44.6	*NA*	39.7
	11	1.60	20.7	1.48	20.7	1.59	27.3	*NA*	20.6
	12	2.22; 1.92	36.8	2.75; 1.10	38.0	4.11	78.4	*NA*	36.6
	13	--	86.2	--	87.2	2.94	42.0	*NA*	85.9
	14	2.70; 1.77	44.6	1.94; 2.45	44.8	1.42; 1.05	39.9	*NA*	44.3
	15	2.09; 2.02	47.7	2.10	48.6	1.91; 2.23	49.0	*NA*	47.5
	16	--	154.5	--	153.8	--	148.4	*NA*	154.3
	17	5.68; 5.04	104.7	5.10; 5.64	105.5	4.90; 5.40	109.5	*NA*	104.5
	18	1.22	28.4	1.42	29.4	1.26	29.1	*NA*	28.2
	19	--	177.2	--	176.1	--	177.4	*NA*	177.0
	20	1.27	15.7	0.99	16.5	1.04	13.3	*NA*	15.4
Glcβ-C19	1′	6.08	95.9	6.23	93.8	6.18	96.4	6.16	95.6
	2′	4.15	74.0	*NA*	*NA*	*NA*	*NA*	*NA*	*NA*
	3′	4.17	79.0	*NA*	*NA*	*NA*	*NA*	*NA*	*NA*
	4′	4.27	71.1	*NA*	*NA*	*NA*	*NA*	*NA*	*NA*
	5′	3.93	79.3	*NA*	*NA*	*NA*	*NA*	*NA*	*NA*
	6′	4.39; 4.30	62.2	*NA*	*NA*	*NA*	*NA*	*NA*	*NA*
Glcβ(1-X)	1″	--	--	5.10	105.8	--	--	--	--
	2″	--	--	*NA*	*NA*	--	--	--	--
	3″	--	--	*NA*	*NA*	--	--	--	--
	4″	--	--	*NA*	*NA*	--	--	--	--
	5″	--	--	*NA*	*NA*	--	--	--	--
	6″	--	--	*NA*	*NA*	--	--	--	--
Glcβ-C13	1‴	5.12	98.0	5.12	99.7	5.01	103.0	5.08	97.6
	2‴	4.14	84.6	--	--	*NA*	*NA*	*NA*	*NA*
	3‴	4.25	78.24	--	--	*NA*	*NA*	*NA*	*NA*
	4‴	4.00	72.2	--	--	*NA*	*NA*	*NA*	*NA*
	5‴	3.88	77.9	--	--	*NA*	*NA*	*NA*	*NA*
	6‴	4.55; 4.19	62.9	--	--	*NA*	*NA*	*NA*	*NA*
Glcβ(1-Y)	1′′′′	5.27	106.8	--	--	5.24	106.4	95.6	104.7
	2′′′′	4.18	77.0	--	--	*NA*	*NA*	*NA*	*NA*
	3′′′′	4.23	78.16	--	--	*NA*	*NA*	*NA*	*NA*
	4′′′′	4.39	71.6	--	--	*NA*	*NA*	*NA*	*NA*
	5′′′′	3.94	78.6	--	--	*NA*	*NA*	*NA*	*NA*
	6′′′′	4.46; 4.41	62.7	--	--	*NA*	*NA*	*NA*	*NA*

^(a)^ NMR spectra recorded in Pyr-*d*_5_, ^(b)^ MeOH-*d*_4_. **10:** stevioside (Y = 2) [79]; **11**: rebaudioside KA (X = 2) [22]; **12:** 12-α-[(2-O-β-D-glucopyranosyl-β-D-glucopyranosyl)oxy]ent-kaur-16-en-19-oic acid β-D-glucopyranosyl ester (Y = 2) [22]; **13:** rebaudioside G (Y = 3) [16] NA: not assigned.

**Table 6:** ^1^H and ^13^C chemical shifts of the compounds **14**–**19**.

Moiety	Position	14 ^(a)^		15 ^(a)^		16 ^(a)^		17 ^(b)^		18 ^(b)^		19 ^(b)^	
		δ_H_	δ_C_	δ_H_	δ_C_	δ_H_	δ_C_	δ_H_	δ_C_	δ_H_	δ_C_	δ_H_	δ_C_
Aglycone	1	2.34; 0.98	39.1	0.78; 1.77	41.2	0.73; 1.73	41.2	0.92; 1.59	39.5	0.87; 1.87	41.4	0.86; 1.87	41.5
	2	2.18; 1.14	20.1	2.22; 1.45	19.9	2.20; 1.43	19.9	1.92; 1.40	18.4	1.41; 1.92	19.8	1.41; 1.94	19.7
	3	2.21; 1.95	37.4	1.03; 2.35	37.3	2.28; 1.79	36.8	2.20; 1.09	37.7	1.06; 2.14	38.8	1.05; 2.17	38.8
	4	--	43.4	--	44.5	--	44.5	--	43.7	--	43.8	--	44.6
	5	1.01	57.9	1.05	57.8	1.03	57.8	1.12	57.1	1.12	58.1	1.12	58.2
	6	2.47; 1.90	22.9	2.46; 1.92	22.6	1.91; 2.50	22.7	1.78; 2.05	19.9	1.84; 1.97	22.6	1.84; 2.04	22.8
	7	1.25; 1.25	42.4	1.30	42.2	1.28; 1.28	42.2	1.57; 1.16	38.8	1.41; 1.54	42.2	1.42; 1.55	42.4
	8	--	44.7	--	43.1	--	43.3	--	33.9	--	43.0	--	43.4
	9	0.86	54.6	0.88	54.5	0.87	54.3	1.09	51.0	0.97	54.7	0.98	55.0
	10	--	40.5	--	40.3	--	40.3	--	38.1	--	39.4	--	40.5
	11	1.60; 1.60	21.4	1.68	21.1	1.68; 1.68	21.1	1.63; 1.45	26.3	1.64; 1.80	21.0	1.65; 1.81	21.1
	12	1.70; 0.73	41.4	2.25; 2.00	37.3	1.02; 2.35	38.9	2.51	41.0	1.52; 1.97	37.9	1.55; 1.98	37.8
	13	--	86.8	--	86.9	--	86.4	--	77.8	--	87.8	--	87.7
	14	2.72; 1.78	45.1	2.66; 1.81	45.0	1.97; 2.74	45.1	4.04	37.7	1.50; 2.27	45.0	1.51; 2.26	45.0
	15	2.04; 2.04	48.3	2.05	48.2	2.06; 2.06	48.0	1.15; 2.51	47.4	2.04; 2.12	48.2	2.04; 2.14	48.4
	16	--	155.2	--	154.7	--	155.0	--	146.7	--	154.5	--	154.4
	17	5.70; 5.07	105.4	5.01; 5.64	105.1	5.10; 5.73	105.4	1.89; 2.11	108.0	4.85; 5.18	105.3	4.84; 5.17	105.2
	18	1.25	28.9	1.25	28.8	1.23	28.8	4.70; 4.84	27.5	1.21	28.6	1.23	28.5
	19	--	178.0	--	177.5	--	177.7	1.24	176.8	--	178.4	--	178.4
	20	1.28	16.3	1.32	16.0	1.31	16.0	0.89	11.9	0.97	16.0	0.98	16.0
Glcβ-C19	1′	6.01	96.5	6.12	96.3	6.12	96.3	5.43	94.2	5.37	95.4	5.34	95.5
	2′	4.09	74.4	4.13	79.8	4.17	74.4	3.39	72.6	3.36	73.8	3.35	74.0
	3′	4.13	79.5	4.18	78.7	3.98	79.5	3.43	77.3	3.46	78.5	3.44	78.4
	4′	4.26	72.0	4.28	71.5	4.33	71.4	3.39	69.7	3.36	70.9	3.45	70.9
	5′	4.02	78.8	3.97	79.2	4.22	79.9	3.39	77.3	3.36	78.2	3.45	77.5
	6′	4.68; 4.31	70.0	4.40	63.4	4.43; 4.57	62.6	3.85; 3.71	61.0	3.68; 3.82	62.2	3.58; 3.61	62.1
Glcβ -C13	1″	5.03	105.9	5.08	98.8	5.19	98.3	4.56	100.5	4.59	97.2	4.61	97.4
	2″	4.00	75.9	4.38	81.3	4.23	84.4	3.65	79.0	3.58	81.5	3.45	82.4
	3″	3.88	79.1	4.15	88.6	4.27	78.6	3.71	86.0	3.55	77.9	3.56	78.1
	4″	4.22	77.8	3.87	71.2	4.45	71.8	3.38	68.8	3.30	71.6	3.25	72.0
	5″	3.88	79.2	3.79	77.9	4.08	77.8	3.34	76.1	3.22	77.5	3.26	77.7
	6″	4.57; 4.38	63.5	4.50; 4.40	63.2	4.51; 4.78	70.3	3.91; 3.68	61.4	3.65; 3.83	62.2	3.63; 3.85	63.0
Glcβ(1-2)	1′′′	5.15	98.7	5.58	105.3	5.30	106.8	4.81	102.2	4.61	104.3	4.57	105.1
	2′′′	4.18	85.4	4.20	76.8	4.11	75.7	3.19	74.6	3.28	75.6	3.27	76.2
	3′′′	3.92	78.7	4.31	74.4	4.23	78.8	3.35	76.5	3.33	77.8	3.36	78.0
	4′′′	4.29	78.9	4.28	72.0	4.28	72.1	3.22	70.6	3.33	71.5	3.30	71.6
	5′′′	4.03	72.8	3.97	79.0	3.91	78.3	3.33	76.6	3.44	77.5	3.25	78.4
	6′′′	4.57; 4.22	63.2	4.40	62.5	4.22; 4.57	63.3	3.87; 3.66	61.9	3.78; 4.10	69.3	3.63; 3.91	63.0
Sugar*(1-X)	1′′′′	5.30	107.6	5.33	105.3	5.14	105.9	4.65	103.1	4.54	104.0	3.61; 3.67	63.8
	2′′′′	4.22	78.8	4.08	76.8	4.06	77.2	3.28	73.9	3.19	74.8	--	105.1
	3′′′′	4.27	71.5	4.20	74.4	4.27	78.9	3.39	76.8	3.46	77.9	4.09	78.8
	4′′′′	4.45	72.1	4.23	72.4	4.12	72.6	3.31	70.1	3.29	71.5	4.00	76.3
	5′′′′	3.97	79.4	4.05	78.8	4.03	78.5	3.37	76.8	3.30	77.8	3.72	83.6
	6′′′′	4.49; 4.38	63.2	4.57; 4.32	62.8	4.22; 4.57	63.3	3.90; 3.66	61.2	3.61; 3.86	62.6	3.72; 3.98	61.6

^(a)^ NMR spectra recorded in Pyr-*d*_5_, ^(b)^ MeOH-*d*_4_. **14**: rebaudioside Y (X = 6) [58]; **15**: rebaudioside A (*Glc, X = 3) [79] **16**: rebaudioside Z (*Glc, X = 6) [31]; **17**: 13-[(2-O-β-D-glucopyranosyl-3-O-β-D-glucopyranosyl-β-D-glucopyranosyl) oxy]ent-hydroxyatis-16-en-19-oic acid -β-D-glucopyranosy ester (*Glc, X = 3) [31]; **18**: 13-[(2-*O*-(6-*O*-β-D-glucopyranosyl)-3-O-β-D-glucopyranosyl-β-D-glucopyranosyl)oxy] kaur-16-en-18-oic acid β-D-glucopyranosyl ester (*Glc, X = 6 as follows Glcβ(1-6)Glcβ(1-2)Glcβ_1_- [65]; **19**: 13-[(2-*O*-β-D-glucopyranosyl-3-*O*-β-D-fructofuranosyl-β-D-glucopyranosyl)oxy] kaur-16-en-18-oic acid β-D-glucopyranosyl ester (*Fru, X = 3) [65]. NA: not assigned.

**Table 7:** ^1^H and ^13^C chemical shifts of the compounds **20**–**23**.

Moiety	Position	20 ^(a)^		21 ^(a)^		22 ^(b)^		23 ^(b)^	
		δ_H_	δ_C_	δ_H_	δ_C_	δ_H_	δ_C_	δ_H_	δ_C_
Aglycone	1	0.77; 1.78	40.8	0.75; 1.77	40.1	0.85; 1.87	41.6	0.85; 1.88	41.5
	2	1.41; 2.20	19.4	1.42; 2.22	19.9	1.44; 1.93	19.8	1.41; 1.94	19.8
	3	1.00; 2.32	38.4	1.89; 2.38	38.9	1.06; 2.17	38.7	1.05; 2.15	38.7
	4	--	44.0	--	44.4	--	44.3	--	44.8
	5	1.02	57.3	1.05	57.8	1.12	58.4	1.12	58.2
	6	1.89; 2.44	22.1	1.98; 2.40	22.6	1.83; 2.08	22.8	1.83; 2.03	22.8
	7	1.28	41.7	1.36; 1.43	42.2	1.43; 1.55	42.5	1.44; 1.55	42.4
	8	--	42.5	--	44.7	--	43.2	--	43.3
	9	0.89	54.0	0.91	54.7	0.98	54.7	0.97	55.0
	10	--	39.8	--	40.2	--	38.8	--	40.7
	11	1.66	20.6	1.68	20.9	1.66; 1.82	21.0	1.63; 1.80	21.0
	12	1.85; 2.28	37.0	2.19; 1.89	38.4	1.46; 2.01	37.6	1.54; 1.99	37.9
	13	--	86.4	--	87.2	--	87.1	--	88.0
	14	1.79; 2.65	44.3	1.74; 2.55	44.7	1.54; 2.27	45.0	1.51; 2.26	45.0
	15	2.03; 2.14	47.7	2.06; 2.14	48.5	2.04	48.3	2.04; 2.14	48.3
	16	--	154.2	--	154.7	--	153.6	--	153.7
	17	4.99; 5.63	104.6	5.20; 5.60	105.4	4.82; 5.11	104.7	4.84; 5.18	105.2
	18	1.20	28.3	1.27	29.0	1.21	28.6	1.20	28.6
	19	--	177.0	--	177.3	--	178.2	--	178.5
	20	1.31	15.5	1.25	16.1	1.00	16.0	0.97	16.0
Glcβ-C19	1′	6.10	95.8	6.16	96.3	5.34	95.7	5.34	95.4
	2′	4.19	73.9	*NA*	*NA*	3.32	73.8	3.35	73.7
	3′	4.10	79.2	*NA*	*NA*	3.44	78.4	3.42	78.2
	4′	4.24	71.0	*NA*	*NA*	3.34	70.9	3.39	70.9
	5′	3.93	78.5	*NA*	*NA*	3.36	78.2	3.52	77.6
	6′	4.39; 4.30	62.1	*NA*	*NA*	3.60; 3.82	62.5	3.76; 4.06	68.8
Xyl(1-6)	1″	--	--	--	--	--	--	4.30	104.6
	2″	--	--	--	--	--	--	3.58	72.2
	3″	--	--	--	--	--	--	3.53	74.0
	4″	--	--	--	--	--	--	3.79	69.1
	5″	--	--	--	--	--	--	3.50; 3.84	66.3
	6″	--	--	--	--	--	--	--	--
Sugar*-C13	1′′′	5.02	97.9	4.96	98.7	4.59	97.8	4.60	97.4
	2′′′	4.22	80.7	*NA*	*NA*	3.59	81.5	3.45	82.4
	3′′′	4.06	88.3	*NA*	*NA*	3.68	87.3	3.54	77.9
	4′′′	3.83	70.6	*NA*	*NA*	3.34	70.9	3.25	71.6
	5′′′	3.72	77.3	*NA*	*NA*	3.30	77.2	3.25	77.9
	6′′′	4.42; 4.02	62.6	--	--	3.60; 3.82	62.3	3.62; 3.83	62.6
Sugar**(1-2)	1′′′′	5.42	105.4	5.54	105.0	4.63	104.0	4.58	105.1
	2′′′′	4.10	75.9	*NA*	*NA*	3.25	73.4	3.27	77.8
	3′′′′	4.12	78.6	*NA*	*NA*	3.42	78.6	3.36	77.9
	4′′′′	4.23	71.2	*NA*	*NA*	3.32	71.1	3.29	71.4
	5′′′′	4.35; 3.65	67.5	*NA*	*NA*	3.36	78.4	3.24	77.7
	6′′′′	--	--	*NA*	*NA*	3.62; 3.80	62.7	3.62; 3.83	62.6
Sugar***(1-3)	1′′′′′	5.26	104.8	5.33	105.1	4.61	105.3	--	--
	2′′′′′	4.01	75.2	*NA*	*NA*	3.54	73.0	--	--
	3′′′′′	4.17	79.0	*NA*	*NA*	3.50	74.3	--	--
	4′′′′′	4.13	71.5	*NA*	*NA*	3.46	69.9	--	--
	5′′′′′	4.02	78.6	*NA*	*NA*	3.62; 3.86	67.6	--	--
	6′′′′′	4.51; 4.26	62.3	*NA*	*NA*	--	--	--	--

^(a)^ NMR spectra recorded in Pyr-*d*_5_, ^(b)^ MeOH-*d*_4_. **20**: rebaudioside F (*Glc, **Xyl and ***Glc) [11]; **21**: rebaudioside R (*Xyl, **Glc and ***Glc) [23]; **22**: 13-[(2-*O*-β-D-glucopyranosyl-3-*O*-β-D-xylopyranosyl-β-D-glucopyranosyl)oxy] *ent*-kaur-16-en-19-oic acid β-D-glucopyranosyl ester (*Glc, **Glc and *** Xyl) [68]; **23**: 13-[(2-O-β-D-glucopyranosyl-β-D-glucopyranosyl) oxy]-kaur-16-en-18-oic acid-(6-O-β-D-xylopyranosyl-β-D-glucopyranosyl) ester (*Glc and **Glc) [37].

**Table 8:** ^1^H and ^13^C chemical shifts of the compounds **24**–**27**.

Moiety	Position	24 ^(b)^		25 ^(b)^		26 ^(b)^		27 ^(a)^	
		δ_H_	δ_C_	δ_H_	δ_C_	δ_H_	δ_C_	δ_H_	δ_C_
Aglycone	1	0.76; 1.66	41.1	0.83; 1.85	40.9	0.85; 1.86	41.6	*NA*	40.8
	2	1.70; 2.11	20.3	1.40; 1.92	19.2	1.40; 1.94	19.8	*NA*	19.4
	3	1.91; 2.15	38.1	1.55; 1.96	37.0	1.05; 2.14	38.9	*NA*	38.5
	4	--	44.8	--	43.5	--	44.8	*NA*	43.9
	5	1.00	58.8	1.07	57.5	1.12	58.3	*NA*	57.5
	6	1.86; 2.11	22.5	1.86; 1.90	21.8	1.84; 2.02	22.7	*NA*	22.0
	7	1.32; 1.62	42.1	1.43; 1.54	41.7	1.41; 1.54	42.5	*NA*	41.8
	8	--	43.1	--	54.0	-	43.2	*NA*	42.2
	9	0.91	54.4	0.98	54.1	0.97	55.0	*NA*	54.1
	10	--	40.2	--	39.0	--	*NA*	*NA*	39.9
	11	1.70	21.1	1.63; 1.80	19.8	1.63; 1.77	21.0	*NA*	20.6
	12	2.68; 1.15	38.1	1.07; 2.27	37.5	1.51; 1.97	38.1	*NA*	38.5
	13	--	86.6	--	87.6	-	88.7	*NA*	87.3
	14	1.70; 2.50	45.1	1.50; 2.24	44.6	1.52; 2.24	44.9	*NA*	43.2
	15	2.10	48.3	2.04; 2.13	47.7	2.03; 2.14	48.5	*NA*	48.7
	16	--	155.1	--	152.5	--	153.2	*NA*	152.9
	17	5.10; 5.72	105.6	4.84; 2.13	104.7	4.83; 5.18	105.1	*NA*	106.1
	18	1.50	29.6	1.25	28.4	1.21	28.6	*NA*	28.2
	19	--	176.0	--	177.3	--	178.4	*NA*	177.1
	20	1.14	17.3	0.93	16.1	0.99	16.1	*NA*	15.2
Glcβ-C19	1′	6.24	94.1	5.60	93.5	5.38	95.5	5.98	96.0
	2′	*NA*	*NA*	3.60	77.7	3.34	73.6	*NA*	*NA*
	3′	*NA*	*NA*	3.57	78.2	3.44	78.4	*NA*	*NA*
	4′	*NA*	*NA*	3.40	70.1	3.34	70.9	*NA*	*NA*
	5′	*NA*	*NA*	3.39	77.4	3.36	78.2	*NA*	*NA*
	6′	*NA*	*NA*	3.70; 3.79	62.3	3.60; 3.82	62.3	*NA*	*NA*
Rha(1-2)	1″	6.40	102.0	5.30	100.9	--	--	--	--
	2″	*NA*	*NA*	3.90	71.0	--	--	--	--
	3″	*NA*	*NA*	3.61	71.1	--	--	--	--
	4″	*NA*	*NA*	3.37	72.7	--	--	--	--
	5″	*NA*	*NA*	3.75	69.3	--	--	--	--
	6″	*NA*	*NA*	1.24	17.1	--	--	--	--
Glcβ-C13	1′′′	5.10	98.5	4.61	96.5	4.56	97.7	5.04	97.7
	2′′′	*NA*	*NA*	3.47	81.2	3.54	80.9	*NA*	*NA*
	3′′′	*NA*	*NA*	3.57	79.2	3.67	87.5	*NA*	*NA*
	4′′′	*NA*	*NA*	3.31	70.7	3.34	70.9	*NA*	*NA*
	5′′′	*NA*	*NA*	3.25	77.2	3.30	77.2	*NA*	*NA*
	6′′′	*NA*	*NA*	3.63; 3.83	62.0	3.60; 3.82	62.3	*NA*	*NA*
Sugar*(1-2)	1′′′′	5.22	107.0	4.60	104.4	4.71	104.0	6.34	104.2
	2′′′′	*NA*	*NA*	3.24	75.0	3.22	75.8	*NA*	*NA*
	3′′′′	*NA*	*NA*	3.35	77.7	3.30	74.0	*NA*	*NA*
	4′′′′	*NA*	*NA*	3.21	70.7	3.02	76.9	*NA*	*NA*
	5′′′′	*NA*	*NA*	3.21	76.5	3.26	81.2	*NA*	*NA*
	6′′′′	*NA*	*NA*	3.64; 3.84	61.8	1.25	18.0	*NA*	18.8
Glcβ(1-3)	1′′′′′′	--	--	--	--	4.63	104.2	4.94	102.1
	2′′′′′′	--	--	--	--	3.24	73.4	*NA*	*NA*
	3′′′′′′	--	--	--	--	3.42	78.6	*NA*	*NA*
	4′′′′′′	--	--	--	--	3.32	71.1	*NA*	*NA*
	5′′′′′′	--	--	--	--	3.36	78.4	*NA*	*NA*
	6′′′′′′	--	--	--	--	3.62; 3.80	62.7	*NA*	*NA*

^(a)^ NMR spectra recorded in Pyr-*d*_5_, ^(b)^ MeOH-*d*_4_. **24**: rebaudioside S [23]; **25**: no trivial or systematic name was assigned (*Glc) [25]; **26**: 13-[(2-O-6-deoxy-β-D-glucopyranosyl-3-O-β-D-glucopyranosyl-β-D-glucopyranosyl)oxy] ent-kaur-16-en19-oic acid β-D-glucopyranosyl ester (*6deoxyGlc) [71]; **27**: rebaudioside C (*Rha) [16].

## Sample Availability

Samples of the compounds described in this review are available from the authors.

## References

[1] Kinghorn A.D., Kinghorn A.D. (2002). Stevia: The Genus Stevia; Medicinal and Aromatic Plants—Industrial Profiles.

[2] Cioni P.L., Morelli I., Andolfi L., Macchia M., Ceccarini L. (2006). Qualitative and quantitative analysis of essential oils of five lines stevia rebaudiana bert. genotypes cultivated in pisa (italy). J. Essent. Oil. Res..

[3] McGarvey B.D., Attygalle A.B., Starratt A.N., Xiang B., Schroeder F.C., Brandle J.E., Meinwald J. (2003). New Non-Glycosidic Diterpenes from the Leaves of Stevia r Ebaudiana. J. Nat. Prod..

[4] Rajbhandari A., Roberts M.F. (1983). The Flavonoids of Stevia Rebaudiana. J. Nat. Prod..

[5] Karaköse H., Jaiswal R., Kuhnert N. (2011). Characterization and quantification of hydroxycinnamate derivatives in stevia rebaudiana leaves by LC-MSn. J. Agric. Food Chem..

[6] Bridel M., Lavielle R. (1931). Sur Le Principe Sucre Des Feuilles de Kaa-He-e (Stevia Rebaundiana B). Acad. Sci. Paris Comptes Rendus.

[7] Kohda H., Kasai R., Yamasaki K., Murakami K., Tanaka O. (1976). New sweet diterpene glucosides from stevia rebaudiana. Phytochemistry.

[8] Kobayashi M., Horikawa S., Degrandi I.H., Ueno J., Mitsuhashi H. (1977). Dulcosides A and B, New diterpene glycosides from stevia rebaudiana. Phytochemistry.

[9] Sakamoto I., Yamasaki K., Tanaka O. (1977). Application of 13C NMR spectroscopy to chemistry of plant glycosides: Rebaudiosides-d and-e, new sweet diterpene-glucosides of stevia rebaudiana bertoni. Chem. Pharm. Bull..

[10] Sakamoto I., Yamasaki K., Tanaka O. (1977). Application of 13C NMR spectroscopy to chemistry of natural glycosides: Rebaudioside-c, a new sweet diterpene glycoside of stevia rebaudiana. Chem. Pharm. Bull..

[11] Starratt A.N., Kirby C.W., Pocs R., Brandle J.E. (2002). Rebaudioside F, A diterpene glycoside from stevia rebaudiana. Phytochemistry.

[12] Andress S. (2008). Agency Response Letter GRAS Notice No. GRN 000252, CFSAN/Office of Food Additive Safety.

[13] Application A540—Steviol Glycosides as Intense Sweeteners. https://www.foodstandards.gov.au/code/applications/pages/applicationa540stevi3096.aspx.

[14] The European Commission (2011). Commission Regulation (EU) No. 1131/2011: Amending Annex II to Regulation (EC) No. 1333/2008 of the European Parliament and of the Council with Regard to Steviol Glycosides. Off. J. Eur. Union.

[15] McQuate R.S. (2010). Agency Response Letter GRAS Notice No. GRN000304, CFSAN/Office of Food Additive Safety.

[16] Ohta M., Morita K., Inoue A., Tamai T., Fujita I., Ohta M., Sasa S. (2010). Characterization of novel steviol glycosides from leaves of stevia rebaudiana morita. J. Appl. Glycosci..

[17] Philippaert K., Pironet A., Mesuere M., Sones W., Vermeiren L., Kerselaers S., Pinto S., Segal A., Antoine N., Gysemans C. (2017). Steviol glycosides enhance pancreatic Beta-Cell function and taste sensation by potentiation of TRPM5 channel activity. Nat. Commun..

[18] Gu W., Rebsdorf A., Anker C., Gregersen S., Hermansen K., Geuns J.M.C., Jeppesen P.B. (2019). Steviol glucuronide, a metabolite of steviol glycosides, potently stimulates insulin secretion from isolated mouse islets: Studies in vitro. Endocrinol. Diabetes Metab..

[19] Geuns J.M.C., Buyse J., Vankeirsbilck A., Temme E.H.M., Compernolle F., Toppet S. (2006). Identification of Steviol Glucuronide in Human Urine. J. Agric. Food Chem..

[20] Chen T.-H., Chen S.-C., Chan P., Chu Y.-L., Yang H.-Y., Cheng J.-T. (2005). Mechanism of the hypoglycemic effect of stevioside, a glycoside of stevia rebaudiana. Planta Med..

[21] Jeppesen P.B., Gregersen S., Rolfsen S.E.D., Jepsen M., Colombo M., Agger A., Xiao J., Kruhøffer M., Orntoft T., Hermansen K. (2003). Antihyperglycemic and blood pressure-reducing effects of stevioside in the diabetic goto-kakizaki rat. Metabolism.

[22] Ibrahim M.A., Rodenburg D.L., Alves K., Fronczek F.R., McChesney J.D., Wu C., Nettles B.J., Venkataraman S.K., Jaksch F. (2014). Minor diterpene glycosides from the leaves of stevia rebaudiana. J. Nat. Prod..

[23] Ibrahim M.A., Rodenburg D.L., Alves K., Perera W.H., Fronczek F.R., Bowling J., McChesney J.D. (2016). Rebaudiosides R and S, Minor diterpene glycosides from the leaves of stevia rebaudiana. J. Nat. Prod..

[24] Perera W.H., Ghiviriga I., Rodenburg D.L., Alves K., Bowling J.J., Avula B., Khan I.A., McChesney J.D. (2017). Rebaudiosides T and U, Minor C-19 xylopyranosyl and arabinopyranosyl steviol glycoside derivatives from stevia rebaudiana (bertoni) bertoni. Phytochemistry.

[25] Purkayastha S., Clos J.F., Prakash I., Yin C.S., Markosyan A. (2019). Additional minor diterpene glycosides from stevia rebaudiana. IOSR J. Appl. Chem..

[26] Perera W.H., Ghiviriga I., Rodenburg D.L., Carvalho R., Alves K., McChesney J.D. (2017). Development of a high-performance liquid chromatography procedure to identify known and detect novel c-13 oligosaccharide moieties in diterpene glycosides from stevia rebaudiana (bertoni) bertoni (asteraceae): Structure elucidation of rebaudiosides V and W. J. Sep. Sci..

[27] Prakash I., Ma G., Bunders C., Charan R.D., Ramirez C., Devkota K.P., Snyder T.M. (2017). A novel diterpene glycoside with nine glucose units from stevia rebaudiana bertoni. Biomolecules.

[28] Prakash I., Hong S., Ma G., Bunders C., Devkota K.P., Charan R.D., Ramirez C., Snyder T.M. (2017). Complete structure elucidation of new steviol glycosides possessing 9 glucose units isolated from stevia rebaudiana. Nat. Prod. Commun..

[29] Ma G., Bechman A., Bunders C., Devkota K.P., Charan R.D., Ramirez C., Snyder T.M., Priedemann C., Prakash I. (2018). New diterpene glycosides from stevia rebaudiana bertoni: Rebaudioside VIII and Rebaudioside IXd. Nat. Prod. Commun..

[30] Prakash I., Ma G., Bunders C., Devkota K.P., Charan R.D., Ramirez C., Snyder T.M., Priedemann C. (2015). A new diterpene glycoside: 15α-hydroxy-rebaudioside m isolated from stevia rebaudiana–Indra prakash. Nat. Prod. Commun..

[31] Perera W.H., Ghiviriga I., Rodenburg D.L., Alves K., Wiggers F.T., Hufford C.D., Fronczek F.R., Ibrahim M.A., Muhammad I., Avula B. (2018). Tetra-Glucopyranosyl diterpene Ent-Kaur-16-En-19-Oic acid and Ent-13(S)-Hydroxyatisenoic acid derivatives from a commercial extract of stevia rebaudiana (bertoni) bertoni. Molecules.

[32] Devkota K.P., Charan R.D., Priedemann C., Donovan R., Snyder T.M., Ramirez C., Harrigan G., Ma G., Prakash I. (2019). Five new ent-atisene glycosides from stevia rebaudiana. Nat. Prod. Commun..

[33] Lee T. (2009). Steviol Glycoside Isomers 2009. U.S. Patent.

[34] Avent A.G., Hanson J., Oliveira B.H. (1990). Hydrolysis of the diterpenoid glycoside, stevioside. Phytochemistry.

[35] Prakash I., Chaturvedula V.S.P., Markosyan A. (2014). Structural characterization of the degradation products of a minor natural sweet diterpene glycoside rebaudioside m under acidic conditions. Int. J. Mol. Sci..

[36] Prakash I., Clos J.F., Chaturvedula V.S.P. (2012). Stability of rebaudioside a under acidic conditions and its degradation products. Food Res. Int..

[37] Chaturvedula V.S.P., Clos J.F., Rhea J., Milanowski D., Mocek U., DuBois G.E., Prakash I. (2011). Minor diterpenoid glycosides from the leaves of stevia rebaudiana. Phytochem. Lett..

[38] Perera W.H., Docampo M.L., Wiggers F.T., Hufford C.D., Fronczek F.R., Avula B., Khan I.A., McChesney J.D. (2018). Endocyclic double bond isomers and by-products from rebaudioside a and stevioside formed under acid conditions. Phytochem. Lett..

[39] Prakash I., Bunders C., Devkota K.P., Charan R.D., Hartz R.M., Sears T.L., Snyder T.M., Ramirez C. (2015). Degradation products of rubusoside under acidic conditions. Nat. Prod. Commun..

[40] Rodenburg D.L., Alves K., Perera W.H., Ramsaroop T., Carvalho R., McChesney J.D. (2016). Development of hplc analytical techniques for diterpene glycosides from stevia rebaudiana (bertoni) bertoni: Strategies to scale-up. J. Braz. Chem. Soc..

[41] Chaturvedula V.S.P., Prakash I. (2011). Utilization of RP-HPLC fingerprinting analysis for the identification of diterpene glycosides from stevia rebaudiana. Int. J. Res. Phytochem. Pharmacol..

[42] Prakash Chaturvedula V.S., Upreti M., Prakash I. (2011). Diterpene glycosides from stevia rebaudiana. Molecules.

[43] Chaturvedula V.S.P., Prakash I. (2013). Hydrogenation of the exocyclic olefinic bond at C-16/C-17 position of Ent-Kaurane diterpene glycosides of stevia rebaudiana using various catalysts. Int. J. Mol. Sci..

[44] Prakash I., Campbell M., Chaturvedula V.S.P. (2012). Catalytic hydrogenation of the sweet principles of stevia rebaudiana, rebaudioside b, rebaudioside c, and rebaudioside d and sensory evaluation of their reduced derivatives. Int. J. Mol. Sci..

[45] Brandle J.E., Telmer P.G. (2007). Steviol glycoside biosynthesis. Phytochemistry.

[46] Ceunen S., Geuns J.M. (2013). Steviol glycosides: Chemical Diversity, metabolism, and function. J. Nat. Prod..

[47] Gerwig G.J., Te Poele E.M., Dijkhuizen L., Kamerling J.P. (2016). Stevia glycosides: Chemical and enzymatic modifications of their carbohydrate moieties to improve the sweet-tasting quality. Adv. Carbohydr. Chem. Biochem..

[48] Ruddat M., Heftmann E., Lang A. (1965). biosynthesis of steviol. Arch. Biochem. Biophys..

[49] Mizukami H., Shiiba K., Ohashi H. (1982). Enzymatic determination of stevioside in stevia rebaudiana. Phytochemistry.

[50] Reich E., Schibli A. (2007). High.-Performance Thin-Layer Chromatography for the Analysis of Medicinal Plants.

[51] Jarne C., Savirón M., Lapieza M.P., Membrado L., Orduna J., Galbán J., Garriga R., Morlock G.E., Cebolla V.L. (2018). High-Performance thin-layer chromatography coupled with electrospray ionization tandem mass spectrometry for identifying neutral lipids and sphingolipids in complex samples. J. AOAC Int..

[52] Jarne C., Cebolla V.L., Membrado L., Galbán J., Savirón M., Orduna J., Garriga R. (2016). Separation and profiling of monoglycerides in biodiesel using a hyphenated technique based on high-performance thin-layer chromatography. Fuel.

[53] Barret L.-A., Polidori A., Bonneté F., Bernard-Savary P., Jungas C. (2013). A new high-performance thin layer chromatography-based assay of detergents and surfactants commonly used in membrane protein studies. J. Chromatogr. A.

[54] Wald J.P., Morlock G.E. (2017). Quantification of steviol glycosides in food products, stevia leaves and formulations by planar chromatography, including proof of absence for steviol and isosteviol. J. Chromatogr. A.

[55] Morlock G.E., Meyer S., Zimmermann B.F., Roussel J.-M. (2014). High-Performance thin-layer chromatography analysis of steviol glycosides in stevia formulations and sugar-free food products, and benchmarking with (ultra) high-performance liquid chromatography. J. Chromatogr. A.

[56] Wölwer-Rieck U. (2012). The leaves of stevia rebaudiana (bertoni), their constituents and the analyses thereof: A review. J. Agric. Food Chem..

[57] Kolb N., Herrera J.L., Ferreyra D.J., Uliana R.F. (2001). Analysis of sweet diterpene glycosides from stevia rebaudiana: Improved HPLC method. J. Agric. Food Chem..

[58] Perera W.H., Ramsaroop T., Carvalho R., Rodenburg D.L., McChesney J.D. (2019). A silica gel orthogonal high-performance liquid chromatography method for the analyses of steviol glycosides: Novel tetra-glucopyranosyl steviol. Nat. Prod. Res..

[59] McChesney J.D., Rodenburg D.L. (2014). Preparative chromatography and natural products discovery. Curr. Opin. Biotechnol..

[60] McChesney J.D., Rodenburg D.L. (2014). Chromatography Methods 2014. U.S Patent.

[61] Minne V.J., Compernolle F., Toppet S., Geuns J.M. (2004). Steviol quantification at the picomole level by high-performance liquid chromatography. J. Agric. Food Chem..

[62] Chaturvedula V.S.P., Kinger K.P., Campbell M.R., Prakash I. (2012). NMR spectral assignments of steviol and steviol monoacetate. J. Chem. Pharm. Res..

[63] Gardana C., Scaglianti M., Simonetti P. (2010). Evaluation of steviol and its glycosides in stevia rebaudiana leaves and commercial sweetener by ultra-high-performance liquid chromatography-mass spectrometry. J. Chromatogr. A.

[64] Chaturvedula V.S.P., Prakash I. (2011). Structure elucidation of three new diterpene glycosides from stevia rebaudiana. Int. J. Phys. Sci..

[65] Chaturvedula V.S.P., Rhea J., Milanowski D., Mocek U., Prakash I. (2011). Two minor diterpene glycosides from the leaves of stevia rebaudiana. Nat. Prod. Commun..

[66] Chaturvedula V.S.P., Meneni S.R. (2017). A new penta β-D-Glucopyranosyl diterpene from stevia rebaudiana. Int. J. Org. Chem..

[67] Chaturvedula V.S.P., Prakash I. (2011). Additional minor diterpene glycosides from stevia rebaudiana. Nat. Prod. Commun..

[68] Prakash I., Chaturvedula V.S.P. (2013). Additional minor diterpene glycosides from stevia rebaudiana bertoni. Molecules.

[69] Chaturvedula V.S.P. (2011). Indra prakash diterpene glycosides from stevia rebaudiana. J. Med. Plants Res..

[70] Chaturvedula V.S.P., Upreti M., Prakash I. (2011). Structures of the novel α-glucosyl linked diterpene glycosides from stevia rebaudiana. Carbohydr. Res..

[71] Chaturvedula V.S.P., Prakash I. (2011). Structures of the novel diterpene glycosides from stevia rebaudiana. Carbohydr. Res..

[72] Chaturvedula V., Rhea J., Milanowski D., Mocek U., Prakash I. (2011). Isolation and structure elucidation of two new minor diterpene glycosdies from stevia rebaudiana. Org. Chem. Curr. Res..

[73] Zimmermann B.F. (2011). Tandem mass spectrometric fragmentation patterns of known and new steviol glycosides with structure proposals—zimmermann—2011—Rapid communications in mass spectrometry. Rapid Commun. Mass. Spectrom..

[74] Perera W.H., Avula B., Khan I.A., McChesney J.D. (2017). Assignment of sugar arrangement in branched steviol glycosides using electrospray ionization quadrupole time-of-flight tandem mass spectrometry. Rapid Commun. Mass. Spectrom..

[75] Tanaka T., Nakashima T., Ueda T., Tomii K., Kouno I. (2007). Facile discrimination of aldose enantiomers by reversed-phase HPLC. Chem. Pharm. Bull..

[76] Wang Y.-H., Avula B., Fu X., Wang M., Khan I.A. (2012). Simultaneous determination of the absolute configuration of twelve monosaccharide enantiomers from natural products in a single injection by a UPLC-UV/MS method. Planta Med..

[77] Perera W.H., Shivanagoudra S.R., Pérez J.L., Kim D.M., Sun Y.K., Jayaprakasha G.S., Patil B. (2021). Anti-Inflammatory, antidiabetic properties and in silico modeling of cucurbitane-type triterpene glycosides from fruits of an indian cultivar of *Momordica charantia* L.. Molecules.

[78] Tangpaisarnkul N., Tuchinda P., Wilairat P., Siripinyanond A., Shiowattana J., Nobsathian S. (2018). Development of pure certified reference material of stevioside. Food Chem..

[79] Steinmetz W.E., Lin A. (2009). NMR studies of the conformation of the natural sweetener rebaudioside A. Carbohydr. Res..

